# Alkaline nucleoplasm facilitates contractile gene expression in the mammalian heart

**DOI:** 10.1007/s00395-022-00924-9

**Published:** 2022-03-31

**Authors:** Alzbeta Hulikova, Kyung Chan Park, Aminah A. Loonat, Mala Gunadasa-Rohling, M. Kate Curtis, Yu Jin Chung, Abigail Wilson, Carolyn A. Carr, Andrew W. Trafford, Marjorie Fournier, Anna Moshnikova, Oleg A. Andreev, Yana K. Reshetnyak, Paul R. Riley, Nicola Smart, Thomas A. Milne, Nicholas T. Crump, Pawel Swietach

**Affiliations:** 1 Department of Physiology, Anatomy and Genetics, University of Oxford, Sherrington Building, Parks Road, Oxford, OX1 3PT UK; 2 Physics Department, University of Rhode Island, 2 Lippitt Rd, Kingston, RI 02881 USA; 3 Unit of Cardiac Physiology, Division of Cardiovascular Sciences, University of Manchester, Manchester, UK; 4 Department of Biochemistry, Advanced Proteomics Facility, University of Oxford, Oxford, UK; 5 MRC Molecular Haematology Unit, Radcliffe Department of Medicine, MRC Weatherall Institute of Molecular Medicine, NIHR Oxford Biomedical Research Centre Haematology Theme, University of Oxford, Oxford, UK

**Keywords:** Acidity, Nucleus, Cardiomyocyte, Contraction, CRIP2

## Abstract

Cardiac contractile strength is recognised as being highly pH-sensitive, but less is known about the influence of pH on cardiac gene expression, which may become relevant in response to changes in myocardial metabolism or vascularization during development or disease. We sought evidence for pH-responsive cardiac genes, and a physiological context for this form of transcriptional regulation. pHLIP, a peptide-based reporter of acidity, revealed a non-uniform pH landscape in early-postnatal myocardium, dissipating in later life. pH-responsive differentially expressed genes (pH-DEGs) were identified by transcriptomics of neonatal cardiomyocytes cultured over a range of pH. Enrichment analysis indicated “striated muscle contraction” as a pH-responsive biological process. Label-free proteomics verified fifty-four pH-responsive gene-products, including contractile elements and the adaptor protein CRIP2. Using transcriptional assays, acidity was found to reduce p300/CBP acetylase activity and, its a functional readout, inhibit myocardin, a co-activator of cardiac gene expression. In cultured myocytes, acid-inhibition of p300/CBP reduced H3K27 acetylation, as demonstrated by chromatin immunoprecipitation. H3K27ac levels were more strongly reduced at promoters of acid-downregulated DEGs, implicating an epigenetic mechanism of pH-sensitive gene expression. By tandem cytoplasmic/nuclear pH imaging, the cardiac nucleus was found to exercise a degree of control over its pH through Na^+^/H^+^ exchangers at the nuclear envelope. Thus, we describe how extracellular pH signals gain access to the nucleus and regulate the expression of a subset of cardiac genes, notably those coding for contractile proteins and CRIP2. Acting as a proxy of a well-perfused myocardium, alkaline conditions are permissive for expressing genes related to the contractile apparatus.

**Supplementary Information:**

The online version contains supplementary material available at 10.1007/s00395-022-00924-9.

## Introduction

Myocardial pH is a tightly regulated variable; nonetheless, shifts in pH have been documented in response to changes in work-load [7, 24], in ischemia [25, 27], maladaptive hypertrophy [16, 57, 75], and heart failure [2, 33]. Processes at the membrane (e.g. cardiac electricity) and in the cytoplasm (e.g. contraction) are acutely sensitive to pH [78], but considerably less is known about the slower-onset actions of pH on gene expression in the cardiac nucleus. The acid–base status of the myocardial interstitium relates to the state of metabolism and vascular perfusion, and could provide useful information for directing appropriate gene expression programmes. An established precedent of how milieu chemistry feeds back on gene expression is myocardial hypoxia [20, 36]. Discrete niches of low oxygen tension have been identified in embryonic compact myocardium [49, 72], expanding into the inner myocardial wall in early postnatal life [72], resulting in localised changes in gene expression. Hypoxia-induced signalling has also been documented in disease states of the adult heart that are associated with metabolic or vascular remodelling [66]. Although acidity and hypoxia are often found to coincide, their signalling pathways are radically different, and it remains to be determined whether changes in pH are capable of acting on genes independently. It is therefore important to know which cardiac genes are selectively pH-responsive, and what physiological context would produce meaningful pH-signals.

The mechanism underpinning a potential pH-sensitivity of gene expression may involve changes in the activity of nuclear proteins. Histidine residues in the DNA-binding domains of specific transcription factors may confer pH-sensitivity (e.g. steroid receptors [23, 44], FOXP2 [5], NFκB [54] and KLF [52]). Another means of transducing a pH-signal onto gene expression could involve acetyltransferases and deacetylases, which are pH-sensitive enzymes that target histones and various other nuclear proteins [48]. However, in order to engage these targets, an acid signal arising in the myocardial interstitium must gain access to the nuclear compartment. Whereas the coupling between extracellular and cytoplasmic pH (pHe/pHc) is well characterised in terms of sarcolemmal acid–base transporters, less is known about the relationship between pHc and nuclear pH (pHn). It is widely assumed that pHn strictly follows pHc because high-conductance nuclear pores are thought to ensure strong coupling between these two compartments [3, 6]. However, the paradigm that pHn merely tracks pHc is challenged [30] by at least two observations as follows: that pH is influenced by Ca^2+^ signalling [71], which differs radically between bulk cytoplasm and nucleoplasm [41, 84], thereby establishing conditions for a nucleus-to-cytoplasm pH gradient (ΔpHnc, defined herein as pHn−pHc); and that intracellular H^+^ diffusivity is too slow [77] to short-circuit ΔpHnc by dissipation through nuclear pores. A non-stoichiometric relationship between pHe and pHn would influence how gene expression responds to acidic microenvironments, and therefore must be mapped.

In this study, we sought to characterise the pH landscape of the postnatal myocardium and assess the impact of pH on gene expression. We reasoned that the developing heart’s dramatic switch from glycolytic to aerobic metabolism [55, 60] and immature coronary vascularization [45] may produce meaningful pH-signals, with the power to influence gene expression. Any such genomic pH-responsiveness that emerges during development may also be retained in later life, and manifest under disease conditions that feature acidosis, such as ischemia. Since the scope of pH to affect gene expression is not intuitive to predict, reductionist studies (e.g. the pH-dependence of TF-DNA binding) are not appropriate for defining the pH-responsive transcriptome. Instead, we opted for an unbiased approach that combines transcriptomics and proteomics, followed by immunoreactivity-based validation of enriched pathways on a gene-by-gene basis. Finally, we delineated the mechanism of pH-sensitivity for the pathway of direct relevance to cardiac function. We also characterised the relationship between nuclear and cytoplasmic pH, and tested the degree to which the cardiac nucleus can regulate the pH of its milieu.

Herein, we provide evidence for pH non-uniformity in the early postnatal myocardium and describe a role for pH in regulating the expression of a small but important subset of cardiac genes that code for elements of the contractile apparatus. We also show how an extracellular acid/base signal is transmitted to the nucleus and therein regulates the expression of selected genes.

## Methods

### Animal procedures

Animal experiments were approved by university ethical review boards and conform to the guidelines from Directive 2010/63/EU. For the cryo-infarct model, rats were anaesthetised by isoflurane (4% for induction, 2% for maintenance in O_2_) delivered by intubation. Pre-and post-operative analgesia was provided (buprenorphine, meloxicam). For the tachypacing model, sheep were anesthetised by inhalation of isofluorane (1–5% in O_2_) for induction and maintenance. Peri-operative analgesia (meloxicam, 0.5 mg/kg) and antibiotics (enrofloxacin 5 mg/kg; oxytetracycline 20 mg/kg) were administered subcutaneously. Animals were euthanised by an approved procedure listed under Schedule 1 of the Animals (Scientific Procedures) Acts 1986: intravenous 200 mg/kg pentobarbitone (sheep), cervical dislocation (mice, adult and neonatal rats).

### pHLIP imaging

CD1 mice were injected subcutaneously (P1/P7) or intraperitoneally (P21 and adult) with Var3 pHLIP fluorescently labelled with Cy5.5 (0.7 nmol/g in sterile PBS) 24 h prior to tissue harvesting under license PPL PF8462746. For ratiometric analyses, Hoechst-33342 (10 mg/kg in sterile PBS) was injected intraperitoneally 2 h prior to tissue collection (P1/P7) and co-injected for P21 and adult. Animals were killed humanely by an approved Schedule 1 method. Kidneys and hearts were excised, rinsed in PBS, blotted dry and mounted in trays of OCT before flash freezing in powdered dry ice. Long-axis sections were cut on a cryostat at 25 µm thickness onto glass slides and stored at – 80 °C. Images were taken on a Leica DM6000 microscope with a motorised stage, using Volocity 6.4.0 (Quorum Technologies) for automatic tiling of the entire heart/kidney section. pHLIP (excitation 683 nm/emission 703 nm) and, where applicable, Hoechst-33342 (excitation 350 nm/emission 461 nm) were imaged sequentially. To prepare ratiometric maps, paired pHLIP and Hoechst images were first analysed for background signal in terms of its mean and standard deviation. Images were background subtracted and resized by a factor of 10 using a bilinear interpolation. Pixelwise ratioing was applied to those pixels that were above a threshold on both channels, defined as two standard deviations above background signal. Finally, the ratiometric images were normalized to the median.

### Neonatal ventricular myocyte culture

Myocyte isolation and culture was performed as described previously [42]. Animals were killed by cervical dislocation according to Schedule 1 of the Animals (Scientific Procedures) Acts 1986. All animal experiments have been approved by Oxford University ethical review boards and conform to the guidelines from Directive 2010/63/EU. Primary neonatal rat ventricular myocytes (NRVMs) were obtained from 1–2 day old Sprague–Dawley rats. Cells were isolated from P1/P2 hearts by enzymatic digestion [90], and a ‘pre-plating’ step was introduced to reduce fibroblast number in the myocyte-containing supernatant. Cells were plated onto fibronectin-coated TC culture dishes or Ibidi slides and cultured in medium (referred to as M2) made of 80% DMEM medium containing 24 mM NaHCO_3_ (D7777, Sigma/Merck) and 20% M199 medium with 26 mM NaHCO_3_ (M4530, Sigma/Merck) and incubated in a 5% CO_2_ atmosphere. Initially, medium was supplemented with 10% horse serum, 5% newborn calf serum and penicillin/streptomycin mixture. Next day, medium was replaced by serum-free M2, modified as required with respect to pH ([HCO_3_^−^] ranging from 1.525 mM to 24.4 mM) [51], pro-hypertrophic ligands, or carnosine (30 mM). For selected experiments, the pH of media was confirmed from Phenol Red absorbance measured on Cytation5 (Biotek) [51]. Pro-hypertrophic ligands included 10 µM phenylephrine in combination with 100 nM ascorbic acid (Sigma/Merck), 100 nM endothelin-1 (Enzo Scientific) dissolved in DMSO, and 1 µM isoprenaline/isoproterenol (Sigma/Merck).

### Cell culture conditions for western blotting and chromatin immunoprecipitation

To assess the effect of p300 inhibitor A485, 2 × 10^6^ NRVM were plated onto 6 cm petri dishes. Next day, cells were serum-starved, followed by incubation in either 3.05 mM (acidic) or 24.4 mM (alkaline) NaHCO_3_ with addition of 0.05% DMSO, or 24.4 mM NaHCO_3_ plus 3 µM A485 (selective p300 inhibitor) for 48 h. Cells then were washed with PBS, and lysates for blotting and ChIP were collected.

### Chromatin immunoprecipitation (ChIP) qPCR

NRVM were fixed for 10 min with 1% formaldehyde (Pierce, Thermofisher Scientific), then sonicated using a Covaris (Woburn, MA) following the manufacturer’s recommendations. Sonicated chromatin was incubated overnight with antibodies against H3K27ac (Diagenode C15410196). Antibody-chromatin complexes were isolated with an equal ratio of Protein A and Protein G dynabeads (ThermoFisher Scientific), then washed three times with RIPA buffer (50 mM HEPES–KOH pH 7.6, 500 mM LiCl, 1 mM EDTA, 1% NP-40, 0.7% Na deoxycholate) and once with Tris–EDTA. Samples were eluted with 50 mM Tris–HCl pH 8.0, 10 mM EDTA, 1% SDS, then treated with RNase A and proteinase K, and crosslinks were reversed at 65 °C overnight. DNA was purified by PCR purification kit (Qiagen). DNA was quantified with SYBR Green Master Mix (ThermoFisher Scientific), relative to input chromatin. Primers used for the *Crip2* promoter were as follows: ACTAGTGTGACCGGGAGTAG (forward); GAGCACGTTAGAGGCAGAAG (reverse).

### Chromatin immunoprecipitation (ChIP) seq

ChIP was conducted on NRVM samples as above, except that sonicated fixed *Drosophila* S2 cells were added to the sonicated NRVM cells prior to immunoprecipitation at a ratio of 1:4. After DNA purification, sequencing libraries were prepared using the NEBNext Ultra II DNA Library Preparation kit (NEB). Samples were sequenced by 40 bp paired-end sequencing with a NextSeq 500 (Illumina). Bioinformatic analysis was conducted as previously described [21]. Briefly, sequencing data quality was checked with fastQC (http://www.bioinformatics.babraham.ac.uk/projects/fastqc/), then reads were trimmed with trim_galore (https://www.bioinformatics.babraham.ac.uk/projects/trim_galore/) and mapped against the rat genome assembly rn6 using Bowtie2 [37]. PCR duplicates were removed using Picard MarkDuplicates (http://broadinstitute.github.io/picard/). Sequence tag (read) directories were generated with the Homer tool makeTagDirectory [28], and the command makeBigWig.pl was used to generate bigwig files for visualisation in UCSC [34], normalizing tag counts to tags per 10^7^. Peak calling was conducted using the Homer tool findPeaks using -style histone, with the input track provided for background correction. Metagene profiles were generated using annotatePeaks.pl. For reference normalization [56], input and IP reads were mapped against the dm6 genome assembly as above, and the ratio of dm6:rn6 reads in input and IP samples were used to scale bigwigs and metaplots.

### Cryo-induced myocardial infarction model

All procedures were carried out under licence PPL30-3322 in compliance with the requirements of the UK Home Office (ASPA1986 Amendments Regulations 2012) incorporating the EU directive 2010/63/EU. Male 6-week-old Sprague–Dawley rats were divided into two groups (cryo-injury or sham surgery). Rats were anesthetized via isoflurane in oxygen (4% for induction, 2% for maintenance) and intubated for ventilation. Rats were maintained on a heated pad with monitoring of temperature, pulse oxygenation and electrocardiogram (MouseMonitor S, Indus Instruments). Following a left thoracotomy and removal of the pericardium, the heart was stabilized by a loose stitch through the apex and myocardial infarction was induced by cryo-injury, via the placement of a 9 mm ø aluminium cylindrical probe cooled to 77 K onto the anterio-apical surface of the left ventricle for 15 s. The chest was closed in layers and the animal allowed to recover. In sham-operated rats, thoracotomy and cardiac exteriorization were performed before the chest was closed. Animals were provided with pre- and post-operative analgesia, in the form of buprenorphine, meloxicam, and with lidocaine to prevent arrhythmia.

### Sheep tachypacing heart failure model

All experiments were conducted in accordance with The United Kingdom Animals (Scientific Procedures) Act, 1986 and European Union Directive EU/2010/63. Local ethical approval was obtained from The University of Manchester Animal Welfare and Ethical Review Board. Heart failure was induced in female Welsh Mountain sheep (~ 18 months) by right ventricular tachypacing as described previously [8, 38]. In brief, anaesthesia was induced and maintained by isoflurane inhalation (1–5% in oxygen). Under aseptic conditions using transvenous approaches a single bipolar endocardial pacing lead was actively fixed to the right ventricular apical endocardium and connected to a cardiac pacemaker (Medtronic Sensia, Medtronic Inc. USA) and buried subcutaneously in the right pre-scapular region. Peri-operative analgesia (meloxicam, 0.5 mg/kg) and antibiotics (enrofloxacin 5 mg/kg or oxytetracycline 20 mg/kg) were administered subcutaneously and animals allowed to recover post-operatively for at least 1 week prior to commencement of tachypacing (210 beats per minute; bpm). Animals were monitored at least once daily for onset of clinical signs of HF including lethargy, dyspnea and weight loss. The designated endpoint for the study was either the onset of signs of HF (dyspnea, lethargy, weight loss) or 49 days of tachypacing. Short-axis fractional area change (measured at mid papillary muscle level) was used as a surrogate for ejection fraction. The mean value in tachypaced animals selected for this study was 0.4, which was significantly weaker contraction compared to sham (0.7).

### Isolation of adult ventricular myocytes

Adult rat, sheep or mouse myocytes were isolated from hearts using enzymatic digestion, and kept in primary culture for no more than 10 h. Myocytes were isolated from Langendorff-perfused rat or mice hearts using a previously published method [18, 26] and from sheep hearts using a previously published method [8, 38].

### Fluorescence pH imaging

Adult myocytes were imaged in superfusion chambers, coated with poly-l-lysine to improve cell adhesion. Neonatal myocytes were imaged as monolayers grown in Ibidi chambers. Live-cell imaging was performed on a Zeiss LSM 700 confocal system. Myocytes or immortalized HCT116 cells were loaded for 10 min with a mixture of 4 µM DNA-binding dye (Hoechst or SYTO family; Sigma/Merck or Thermo-Fisher Scientific) and 20 µM 5-(and-6)-carboxySNARF-1 acetoxymethyl ester (cSNARF1-AM, ThermoFisher Scientific). For in situ measurements under incubation conditions, myocytes were imaged in a mobile incubator (Ibidi, Germany) mounted on the Zeiss microscope, kept at 37 °C, 93% humidity and 5% CO_2_ and imaged after 1 h. For measurements under superfusion, solutions at 37 °C were delivered to myocytes, and recordings were made once the steady-state was attained (> 10 min). Hoechst 34,580 fluorescence was excited at 405 nm and measured at 440 and 490 nm; cSNARF1 fluorescence was excited at 555 nm and measured at 580 and 640 nm. Image analyses measured pHn from nuclear regions and pHc in cytoplasmic regions surrounding nuclei. Hepes-buffered superfusates contained 135 mM NaCl, 4.5 mM KCl, 1 mM MgCl_2_, 1 mM CaCl_2_, 11 mM glucose, 20 mM Hepes titrated to pH 7.4. Carbonic-buffered superfusates were modified to contain 125 mM NaCl and NaHCO_3_ in place of Hepes and were bubbled with 5% CO_2_. Where indicated, drugs were added to superfusates. Pacing was delivered by field stimulation at 0.8, 2 or 4 Hz to load Ca^2+^ stores. To produce Ca^2+^-free superfusates, CaCl_2_ was replaced with 0.5 mM EGTA. To produce Na^+^-free superfusates, NaCl was replaced with *N*-methyl-d-glucamine (NMDG). For acetate-containing solutions, NaCl was replaced with an equimolar amount of NaAcetate. Osmolality of all solutions was set to 300 mOsm and adjusted by reduced [NaCl] if required. All salts were purchased from Sigma/Merck.

### Immunofluorescence

NRVMs (60,000 cells/well) were plated on fibronectin-treated slides with a removable 12-well silicone chamber (Ibidi). Protocol 1: To image CRIP2, ssTnI (*Tnni1*) or glucose-6-phosphate dehydrogenase (G6PDH), cells were fixed with 4% paraformaldehyde in PBS and then permeabilized with 0.2% Triton X-100 for 15 min. Protocol 2: To image sarcolemmal NHE1, cells were first permeabilized with 0.1% Triton X-100 for 1 min at room temperature. To image nuclear NHE1, cells were permeabilized [65] with 0.5% Triton X-100 for 30 min at 4 °C, followed by 1 h incubation with 188 units of DNaseI at 37 °C. Permeabilized cells were then fixed with 4% paraformaldehyde in PBS (Sigma/Merck) for 15 min at room temperature.

After blocking with 3% BSA in PBS for 1 h at RT, cells were incubated with rabbit primary antibodies against NHE1 (Millipore), lamin A/C (CST), CRIP2, ssTnI or G6PDH (1:100, Proteintech) overnight at 4 °C. Cells were then washed 4 × 5 min with 0.1% PBS-Tween 20 and incubated with goat anti-rabbit IgG (H + L) conjugated with Alexa Fluor Plus 488 (Thermo Fisher Scientific). Cell nuclei were co-stained with Hoechst-33342 (Thermo Fisher Scientific) and washed again 4 × 5 min with 0.1% PBS-Tween 20, mounted using ProLong Gold Antifade Mounting medium (ThermoFisher Scientific) and scanned using Zeiss 700 LSM confocal.

### ELISA

NRVMs were lysed with RIPA buffer (CST) for 10 min at 4 °C and centrifuged at 17,000 rpm at 4 °C for 20 min. Supernatant was then used to measure the total protein content by Pierce BCA Protein Assay (ThermoFisher Scientific). Equal amounts (typically 30 µg) of total protein/well were pipetted into 96-well high-binding ELISA microplate (Greiner Bio-One) and topped up to 50 µl with PBS and left to bind overnight at 37 °C. Next day, the plate was washed 2 × with PBS and blocked with 10% FCS in 0.05% PBS-Tween 20 (PBS-T) for 2 h at RT. Samples were incubated for 1 h at RT in the presence of rabbit polyclonal primary antibody against cTnT (Proteintech) or mouse monoclonal Ab against cTnT (ThermoFisher Scientific), rabbit polyclonal Abs against ssTnI (Proteintech), cTnI (Proteintech), CRIP2 (Proteintech or Novus), myosin-7, mouse monoclonal Ab against myosin-6 (Novus) and β-actin-HRP conjugated mouse monoclonal Ab (Proteintech) diluted in blocking buffer. The plate was then washed 4 × with PBS-T and incubated with HRP conjugated goat anti-rabbit IgG (H + L) or goat anti-mouse IgG (H + L) secondary antibody with dilution of 1:5,000 in blocking buffer (Thermo Fisher Scientific) for 1 h at RT and washed again 4 × with PBS-T. Signal was developed with OPD and absorbance was measured at 490 nm using Lx100 plate reader (Biotek) or Cytation5 (Biotek). Measurements were performed on four independent isolations.

### PAGE and immunoblotting

Following treatment, NRVMs cells were lysed in RIPA lysis buffer (Cell Signalling Technologies) with inhibitors of proteases and phosphatases (Roche) for 10 min and stored at − 20 °C until needed. To fractionate lysates, cells were trypsinized, pelleted by centrifugation and soluble/cytoplasmic and residual/nuclear fractions were prepared using Pierce NE-PER Nuclear and Cytoplasmic Extraction Reagent (Thermo Fisher Scientific). The CERI:CERII:NER ratio was 200 µl:11 µl:130 µl. Thawed lysates were spun down at 17,000 rpm for 20 min and supernatant was used for estimation of protein content by BCA assay (Pierce, ThermoFisher Scientific). Lysate was then mixed with beta-mercaptoethanol containing 4 × Laemmli sample buffer (Bio-Rad) and loaded onto 10% polyacrylamide gel and proteins were separated using Mini-PROTEAN system from Bio-Rad. Protein separation was stopped when the 10-kDa marker of Precision Plus Protein Dual Color (Bio-Rad) mixed with Magic Mark (ThermoFisher Scientific) reached the bottom of the gel and 15 kDa marker was still visible in the gel. Separated proteins were then blotted onto PVDF membrane (Bio-Rad) for 2 h at 200 mA. The PVDF membrane was blocked in 5% milk in 0.1% PBS-T for 1 h at RT. Then, membranes were incubated with primary antibodies added to the fresh blocking solution overnight at 4 °C. Primary rabbit antibodies used were against CRIP2 (Crip2, Proteintech or Novus), ssTnI *(Tnni1)*, cTnI *(Tnni3)*, cTnT *(Tnnt2)*, myosin-3 *(Myh3)*, myosin light chain kinase 3 (Proteintech), myosin-6 (*Myh6*), myosin-7 (*Myh-7*), myosin light chain 2 (Novus), mouse monoclonal antibodies against lamin A/C (LMNA/C, CST), vimentin (VIM, CST), α-actinin (ACTN2, Proteintech) and HRP-conjugated primary antibodies against GAPDH and β-actin (GAPDH and ACTB, Proteintech) in dilutions recommended by manufacturer. Next day, membranes were washed in 0.1% PBS-T for 30 min and incubated with respective secondary antibodies in blocking solution for 1 h at RT and developed on X-ray film or digitally using Bio-Rad chemidoc. HRP-conjugated goat anti-rabbit and mouse IgG (H + L) secondary antibodies were used according to manufacturer’s instructions (Thermo Fisher Scientific).

### Whole-exome RNAseq

A biological repeat is considered as a yield of neonatal rat ventricular myocytes (NRVMs) isolated from 10 to 12 P1 pups, which is then split among different pH groups. 2 × 10^6^ NRVM cells were plated into 6 cm fibronectin-coated Petri dishes and treated as described earlier. Total RNA was extracted using Quick-RNA Miniprep Kit (Zymo Research) following manufacturer’s instructions and eluted into nuclease-free water (Qiagen). The A260/A230 ratios were measured using Nanodrop and Spectrostar spectrophotometers. RNA integrity (RI) was estimated using Agilent RNA 6000 Nano Kit and measured by 2100 Bioanalyzer (Agilent). RI number of all samples was 9.9–10. RNA concentration was estimated by QuantiFluor RNA System (Promega) and fluorescence was measured using Cytation5 (Biotek). Three sets of samples with a complete range of five test-pH levels were used for whole-exome RNA sequencing by HiSeq4000_75 PE System (Illumina) in Oxford Genomics centre. Total RNA per sample was 3 µg (100 ng/µl, 30 µl). The mRNA fraction was selected from the total RNA provided before conversion to cDNA. Second-strand cDNA synthesis incorporated dUTP. The cDNA was end-repaired, A-tailed and adapter-ligated. Prior to amplification, samples underwent uridine digestion. The prepared libraries were size-selected, multiplexed and quality-checked before paired end sequencing over one lane of a flow cell. Data were then aligned to the reference and quality checked. Sequence alignment was performed by RNA STAR against the *Rattus norvegicus* rn5 genome. DEGs were identified by the DESeq2 package [43] (version 1.28.1) in R. The model used for testing had multiple levels (five) of the condition (pH).

### Label-free mass spectrometry of in-solution digested samples

5.10^6^ NRVM cells were plated onto 10 cm fibronectin-coated Petri dishes. After the 48-h incubation in serum-free M2 medium, cells were trypsinized, pelleted and fractionated into soluble and residual fractions using Pierce NE-PER Nuclear and Cytoplasmic Extraction Reagent containing Halt cocktail of inhibitors of proteases and phosphatases (Thermo Fisher Scientific). Samples were split in half. One was used for quality control and to measure total protein content by Pierce BCA assay (Thermo Fisher Scientific), and the other was used for proteomics. Three sets of samples at two levels of pH were used for label-free LC–MS/MS proteomic analysis at TDI Mass Spectrometry Laboratory of University of Oxford. DAPs were identified by the DEP package [87] (1.10.0) in R. Missing values can be problematic for proteomic analysis, and were imputed by a left-censored deterministic (minDet) method. This routine applies to proteins that fall below the detection level and is deterministic, and therefore yields the same outcomes with each run.

### Immunoprecipitation of p300

HEK293.T cells were transfected with plasmids encoding FLAG-myocardin and HA-p300. Both expression vectors were a kind gift from Eric Olson (UT Southwestern, USA) and have been described previously [10, 11, 79]. Cells were placed in serum-free media prior to transfection with complexes containing Lipofectamine 3000 reagent (Thermo Fisher Scientific) and plasmid DNA. 24 h after transfection, medium was replaced to change pH to 6.4, 6.9 and 7.4. After 4 h incubation, cells were harvested in lysis buffer (50 mM Tris HCl, pH 7.4, 150 mM NaCl, 1 mM EDTA, 1% Triton X-100 and complete protease inhibitors (Roche Applied Science)). Cellular debris was removed by centrifugation at 12,000 g for 10 min and FLAG-myocardin was precipitated with ANTI-FLAG M2 Affinity Gel (Sigma-Aldrich). Immunoprecipitated protein samples were resolved by SDS-PAGE and transferred to PVDF membranes (Bio-Rad). Membranes were immunoblotted with anti-acetyl Lysine antibody (Abcam) and anti-DYKDDDDK Tag antibody (Cell Signalling Technology) and proteins were visualised with a chemiluminescence detection system (Chemidoc (Bio-rad)).

### Luciferase assays

HEK293.T cells were transfected with plasmids encoding FLAG-myocardin and HA-p300, Renilla luciferase (Promega) and *Tnnt2* firefly luciferase reporter as described above. The *Tnnt2* firefly luciferase reporter was a kind gift from Oliver Mueller (UKSH, Germany) and encodes the Firefly luciferase gene under the control of the 544-bp human *Tnnt2* promoter as previously described [83]. Following transfection and incubation of cells with different pH media, a dual luciferase assay was performed (Promega) using a luminometer according to manufacturer’s instructions.

### Statistics

Statistical testing was performed with hierarchical analysis [67]. RNAseq and proteomics data were analysed by the DESeq2 and DEP packages in R, respectively.

## Results

### Characterising the pH landscape of the early postnatal myocardium

**Fig. 1:**
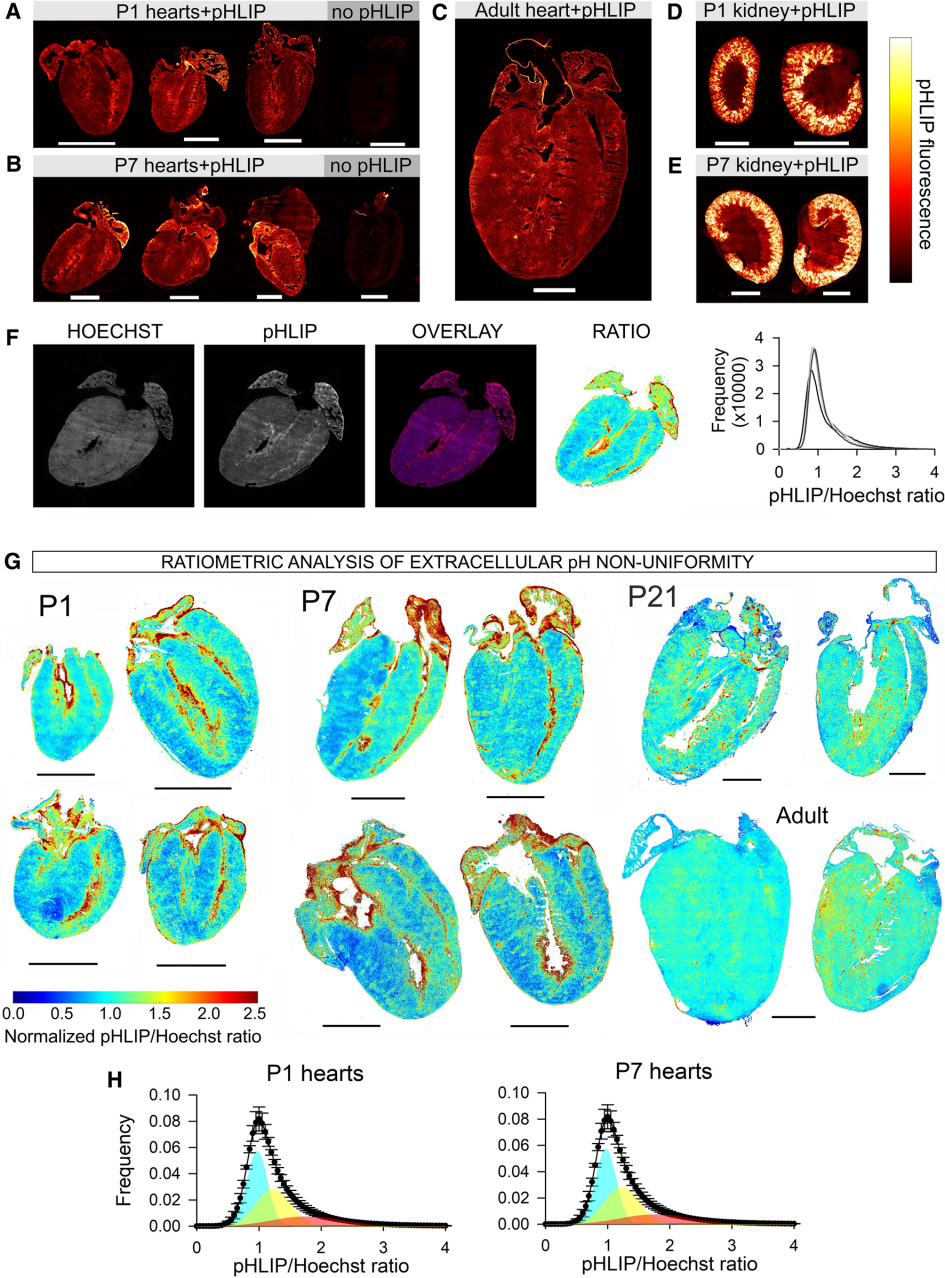
The early postnatal myocardium maintains significant non-uniformity of extracellular pH that dissipates in later life. **A** P1 mice were injected with Cy5.5-pHLIP and hearts were excised one day later (controls received no injection). Images show fluorescence in coronal sections. Experiments were also performed on P7 (**B**) and adult (**C**) mice. **D** Fluorescence images of coronal sections of kidneys from P1 and **E** P7 mice. **F** Ratiometric imaging; hearts were dually stained with Cy5.5-pHLIP and Hoechst-33342. Ratiometric map shows pHLIP/Hoechst fluorescence. Frequency histogram of ratio obtained from 5 slices through the mid-luminal region of the heart. **G** Ratiometric images from P1, P7, P21 and adult hearts. Acidic niches (high ratio) are evident in early life. Scale bar 1.6 mm. H Frequency histograms of ratio from P1 (*N* = 6) and P7 (*N* = 6) are positively skewed and best fitted to three Gaussian distributions

To seek evidence for myocardial pH non-uniformity at the necessary spatial resolution, a fluorescence imaging approach was used. Mice at different stages of early life were injected with Cy5.5-conjugated pH-Low Insertion Peptide (pHLIP), a peptide that inserts into biological membranes exposed to acidic extracellular environments [68]. Thus, Cy5.5-pHLIP fluorescence indicates regions of low extracellular pH (pHe). To allow adequate tissue distribution and insertion of the peptide, hearts were harvested for sectioning one day after pHLIP injection. In postnatal day 1 (P1) and P7 hearts, pHLIP-Cy5.5 fluorescence was strongest in the inner myocardial wall (IMW) (Fig. 1A, B). This signal related to pHLIP insertion, rather than tissue autofluorescence, because non-injected controls emitted negligible fluorescence for the same settings. This non-uniformity of pHLIP distribution was not apparent in adult hearts (Fig. 1C). As confirmation that pHLIP had been injected into animals effectively, and is responding appropriately in vivo, a strong signal was detected in the kidney which is noted for its large pH gradients [86] (Fig. 1D, E).

To confirm that the observed differences in pHLIP distribution relate to pH rather than an artefact of variation in cell density, perfusion and dye penetrance, we implemented a ratiometric imaging approach. As a contrast to pHLIP, we used Hoechst-33342, which is spectrally resolvable from Cy5.5 and stains cells according to proximity to blood capillaries. Mice received the injection of Hoechst-33342 before harvesting tissues (two hours prior for P1 and P7, overnight for P21 and adult mice). Cardiac sections were imaged sequentially for pHLIP and Hoechst fluorescence to obtain a ratiometric image (Fig. 1F). Local variations in cell density or dye penetrance affect both pHLIP and Hoechst signals and, therefore, cancel-out in the ratiometric image. A high pHLIP/Hoechst ratio is thus attributable to local acidosis. Ratiometric images obtained for P1 and P7 hearts indicated considerable pHe gradients, with notable acidity in the IMW (Fig. 1G). In contrast, the ratiometric index of pHe was more uniform in P21 and adult (12–14 week) hearts (Fig. 1G; see Fig. S1 for pHLIP images). We, therefore, provide evidence for substantial pHe gradients in early postnatal hearts, which dissipate in later life. The occurrence of acidic niches coincides with major changes in metabolism and vasculature [45, 55, 60], and under these conditions, pHe may act as a physiological signal.

### Identifying pH-responsive genes and pathways

**Fig. 2:**
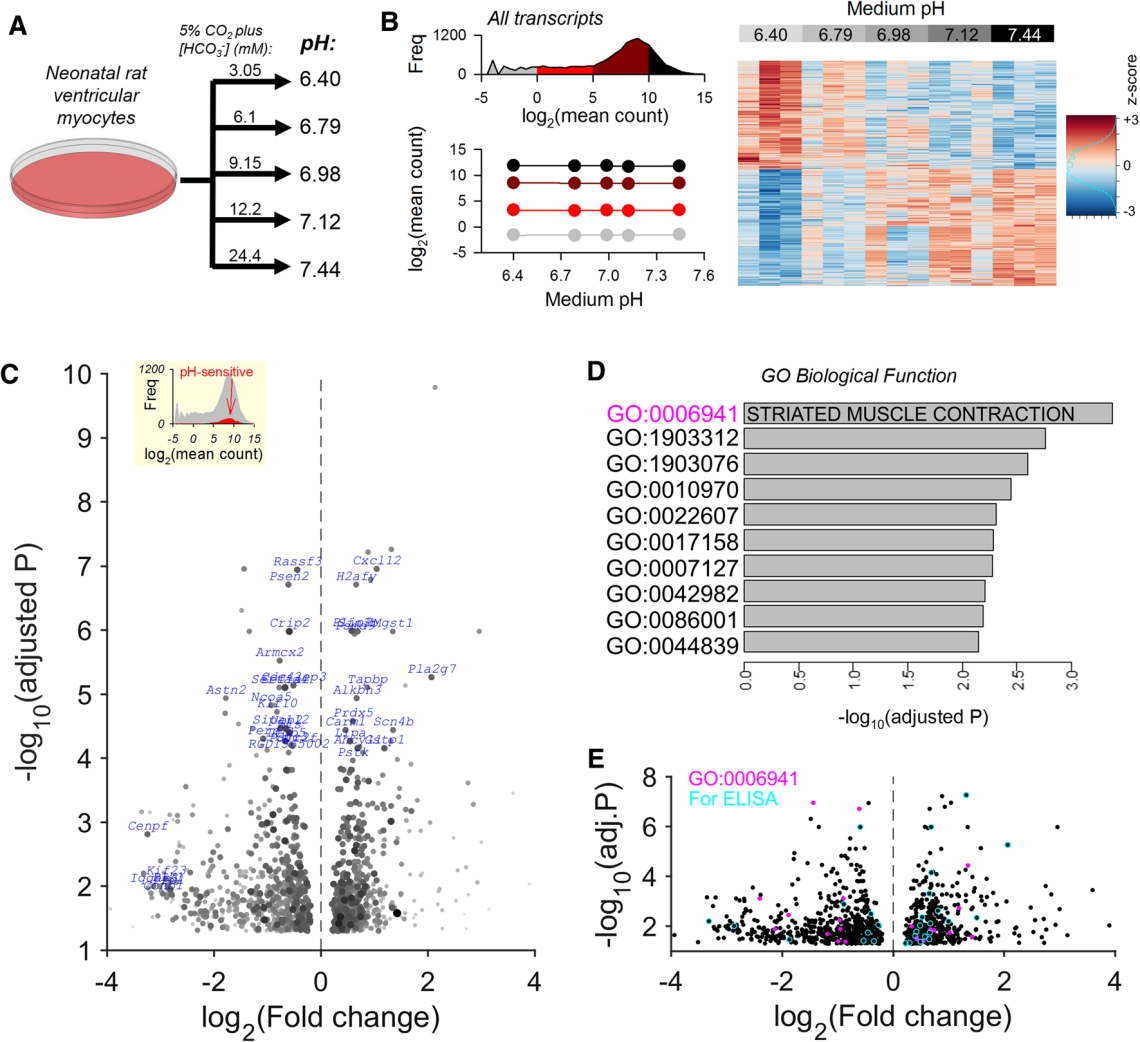
Transcriptomics identify a subset of pH-responsive cardiac genes and pathways. **A** Protocol for determining the pH-responsive transcriptome in neonatal ventricular myocytes (NRVMs). Lysates were collected after 48 h of culture in serum-free medium at one of five pH levels. **B** Analysis of RNAseq performed on total RNA isolated from NRVMs. Histogram of transcripts by expression level, stratified into four groups according to abundance, demonstrating no overall trend in gene expression. Heatmap of differentially expressed genes (DEGs) analysed by DESeq2 using a model with multiple levels of a condition (pH). **C** Volcano plot of pH-responsive DEGs. Genes in blue are a selection of DEGs with a high combination of significance and fold-change, and abundance greater than the median of all DEGs. Fold-change relates to the ratio of signal at acidic and alkaline pH. **D** Gene enrichment analysis identified gene ontology (GO) biological functions associated with pH-responsiveness. GO:0006941: striated muscle contraction; GO:1903312: negative regulation of mRNA metabolic process; GO:1903076: regulation of protein localization to plasma membrane; GO:0010970: transport along microtubule; GO:0022607: cellular component assembly; GO:0017158: regulation of calcium ion-dependent exocytosis; GO:0007127: meiosis I; GO:0042982: amyloid precursor protein metabolic process; GO:0086001: cardiac muscle cell action potential; GO:0044839: cell cycle G2/M phase transition. **E** Volcano plot of pH-responsive DEGs indicating genes of the “striated muscle contraction” ontology (magenta) and genes selected for verification by ELISA inb subsequent experiments (cyan)

To test whether differences in pHe, observed in early postnatal hearts, could affect gene expression, experiments were performed on neonatal rat ventricular myocytes (NRVMs) isolated from P1/P2 pups and cultured over a range of pH (6.40–7.44), prepared according to recently published guidelines [51] (Fig. 2A). Cells were incubated for 48 h which is deemed sufficient to evoke measurable changes in gene expression. Oxygen tension was atmospheric to eliminate hypoxia-specific responses that would often coincide with acidosis in vivo. Additionally, cells were serum-deprived to eliminate possible effects of pH on the transduction of surface-binding ligands. At the end-point, RNA was isolated from cells and analysed by RNAseq whole-exome transcriptomics (for quality-control, see Table S1 and Fig. S2). There was no evidence for a non-specific or generalized pHe-sensitivity among transcripts grouped by abundance. The DESeq2 package, taking a cut-off false discovery rate (FDR)-adjusted *P*-value of 0.05, identified 626 genes that were downregulated as pHe decreased, and 583 genes that showed the opposite response (Fig. 2B; Table S2). These pHe-sensitive differentially expressed genes (pH-DEGs) represented 7% of all transcripts, indicating a highly targeted action. Fibroblasts are a common contaminant in NRVM culture, and a pH-dependent expansion of the former compartment could erroneously identify fibroblast-specific genes as pH-DEGs; however, five fibroblast markers did not change across the tested range of pH (Fig. S3). The volcano plot shown in Fig. 2C highlights, in blue, pH-DEGs with a high combination of fold-change and adjusted probability. Gene Ontology (GO) Enrichment analysis identified “striated muscle contraction” as the most significantly affected biological function (Fig. 2D). The genes belonging to this gene ontology are highlighted in the volcano plot (Fig. 2E). Also highlighted in this plot are genes selected for validation experiments at protein level using ELISA.

### Identifying differentially abundant proteins that are concordant with pH-responsive transcripts

Not all pH-DEGs are expected to manifest a matching pH-dependence at the level of protein abundance. Indeed, a discordance between the transcript-level and protein-level response to pH can be demonstrated for a selection of pH-DEGs tested by ELISA (Fig. 2E: *Cat*, *G6pdh*, *Ahcyl1* and *Ctnnb1*; Fig. S4). To determine which of the proteins align most closely with their transcripts in terms of pH-dependence of expression, an unbiased approach of label-free mass spectrometry was used. NRVMs were cultured for 48 h at either pH 6.40 or 7.44 and lysates were separated into a ‘soluble’ fraction (*F*_S_) enriched with cytoplasmic proteins, and a ‘residual’ fraction (F_R_) enriched in nuclei and cytoskeletal/contractile elements tethered to organellar membranes. Fractionation increases the likelihood of detecting pHe-sensitive gene-products in compartments of low protein abundance, as these could otherwise become obscured by protein-rich compartments. An additional benefit of fractionation is that it can also test for a pH-dependent re-distribution of proteins.

**Fig. 3:**
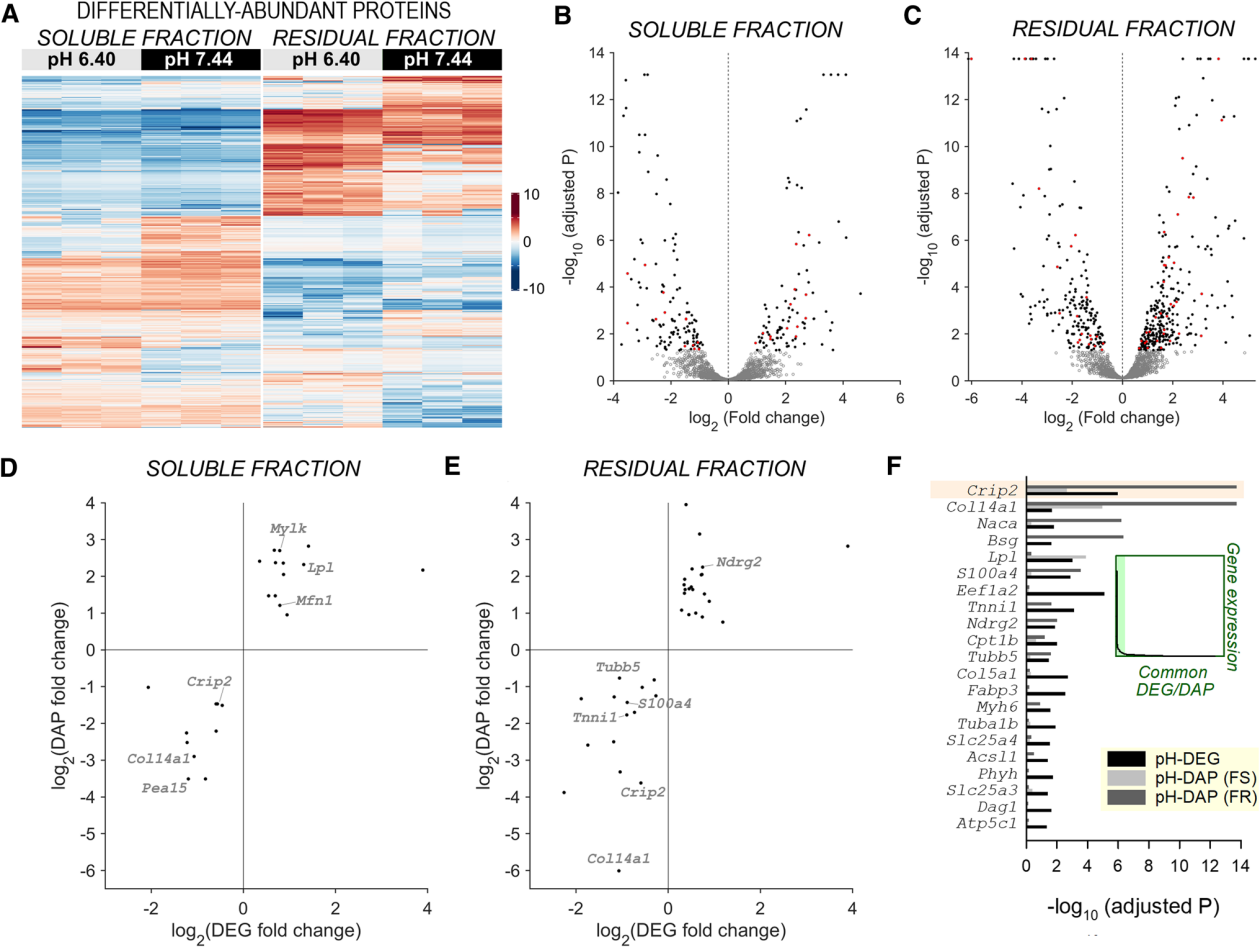
Unbiased proteomic experiments verify the most pH-responsive proteins. **A** Proteomic analysis by label-free mass spectrometry of fractionated NRVM lysates prepared after 48 h of culture in serum-free medium at pH 6.40 or 7.44. Heatmap shows differentially abundant proteins (DAPs). **B** Volcano plot of DAPs in the soluble fraction and **C** residual fraction. DAPs indicated in red were also identified as DEGs by RNAseq. **D** DAPs in the soluble fraction and their corresponding DEGs that showed a coordinated response to pH. Size of circle is proportional to the log_2_ of the mean expression level of corresponding DEG. **E** Analysis repeated for DAPs in the residual fraction. **F** Significance (adjusted *P*-value) for the most abundant pH-responsive DAPs/DEGs, ranked by transcript abundance

In total, 2719 unique proteins were identified, including 2265 proteins in fraction F_S_, 1557 in F_R_ and 1103 proteins present in both fractions (Fig. 3A; Table S3). Of these, 496 proteins in soluble fraction F_S_ (22%) and 211 proteins in residual fraction F_R_ (14%) were determined to be significantly pHe-sensitive by the DEP package, taking a 0.05 FDR-adjusted p-value cut-off and implementing left-censored deterministic minimal imputation approach for missing values (Fig. 3B, C). Overall, 54 pH-DEGs were confirmed to be among the most pH-responsive differentially abundant proteins (pH-DAPs; i.e. ~ 8% of all differentially abundant proteins identified). These pH-DAPs included troponin subunits, determined to be significantly downregulated at low pH, in line with gene enrichment analysis. There was strong concordance in terms of the direction of response between pH-DEGs and the corresponding pH-DAPs (*P* = 3.4 × 10^–5^ in *F*_S_ and *P* = 0.012 in F_R_; Fisher’s exact test; Fig. 3D, E; Table S4). CRIP2 coded by the *Crip2* gene was among the pH-sensitive DAPs and DEGs and scored highly in terms of significance (adjusted P; Fig. 3F).

### Validating pH-responsive genes using immunoreactivity assays

**Fig. 4:**
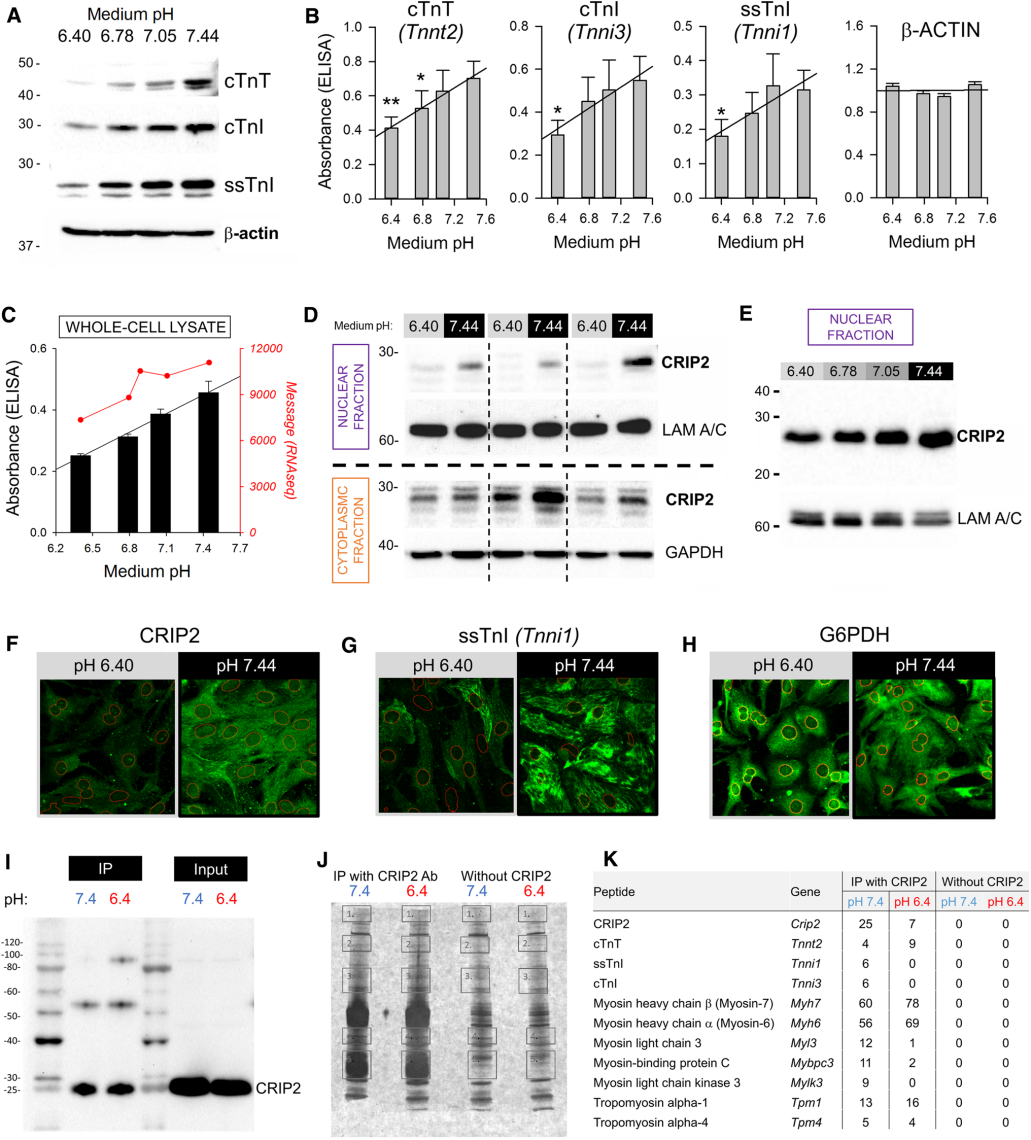
Validating the pH-sensitivity of troponin and Crip2 genes. **A** Western blot of whole-cell lysates collected from NRVMs after 48 h of culture in serum-free medium at one of four test pH levels for cardiac troponin-T (cTnT; *Tnnt2*), cardiac troponin-I (cTnI; *Tnni3*) and slow skeletal troponin-I (ssTnI; *Tnni1*). β-actin was re-developed using the same membrane as that used for ssTnI. **B** ELISA absorbance for cTnT, cTnI and ssTnI, and β-actin as a function of pH, normalized to mean signal (average from 4 isolations). **P < 0.01 and *P < 0.05 by ANOVA. **C** CRIP2 protein quantified by whole-cell ELISA, showing similar pH-dependence to transcript level (4 repeats). **D** Western blot of whole-cell lysates collected from NRVMs after 48 h of culture in serum-free medium at either pH 6.4 or 7.44. Each pair represents an independent isolation (i.e. biological repeat). Fractionated lysates showing pH-sensitivity of CRIP2 in the nucleus and cytoplasm, using lamin A/C and GAPDH as loading controls. **E** CRIP2 western blot of nuclear fractions of NRVM lysates confirm robust pH-responsiveness. **F** Immunofluorescence imaging of NRVM monolayers for CRIP2, **G** ssTnI (a pH-responsive DEG/DAP) and **H** G6PDH (a pH-insensitive protein). Red outlines indicate nuclear regions (Hoechst-33342). (I) Blot for NRVM lysates prepared after immunoprecipitation with CRIP2 antibody, following incubation at pH 6.4 or 7.4. IP blot compared to input. **J** Silver-stained gel produced from CRIP2 immunoprecipitation, highlighting gel areas selected for mass spectrometry. **K** Results of mass spectrometry analysis, highlighting proteins involved in contraction. Only proteins that were absent in the negative control (without CRIP2 antibody) but present in the IP are listed

Enrichment analysis identified “striated muscle contraction” as a biological pathway that responds to pH changes. This gene ontology includes troponin subunits, which are particularly relevant to early postnatal development because of the switch between skeletal and cardiac isoforms [58, 59], specifically a change from slow-skeletal (ss) TnI (*Tnni1*) and ssTnT (*Tnnt1*) expressed in the embryonic stage, to cardiac (c) TnI (*Tnni3*) and cTnT (*Tnnt2*) which then persists in adulthood. Validation of the pHe-sensitivity of cTnT*,* ssTnI and cTnI genes was sought by western blot (Fig. 4A; Fig. S5) and ELISA (Fig. 4B). The results revealed a consistent pHe-dependence of immunoreactivity in all three troponin proteins tested, confirming transcriptomic analyses. At protein level, certain myosin-related components of the contractile apparatus, including myosin-3 (coded by *Myh3*) and myosin heavy chains α and β (*Myh6* and *Myh7*), were downregulated by acidic pH, in line with the effect on troponin isoforms (Fig. S6; see Fig. S7 for quantification). In contrast, isoforms such as myosin light chain 2 (*Myl2*) and myosin light chain kinase (*Mylk3*), were found to be largely pH-insensitive (Fig. S8).

Among the most abundant transcripts in the heart, the gene with the strongest pH-sensitivity at message and protein level was *Crip2*, a pleiotropic transcriptional regulator and cytoplasmic adaptor protein [15, 69, 89], notable for its high expression in the heart (in the top 1%). Whilst little is known about the cardiac biology of *Crip2*, our findings highlight this gene and its protein as a useful marker of acidity. To confirm that the pH-dependence of *Crip2* expression produces pH-dependence at protein level, we performed ELISA (Fig. 4C), western blotting (Fig. 4D, E; see Figs. S9, S10 for uncropped gels) and immunofluorescence (Fig. 4F). For western blotting, lysates were fractionated into cytoplasmic and nuclear, using GAPDH and LAM A/C, respectively, as the loading controls. The results confirmed that CRIP2 levels are robustly pHe-sensitive in both the nuclear and cytoplasmic fraction of NRVMs. String analysis indicated an association between CRIP2 and another pH-responsive protein, ssTnI, which was verified by immunofluorescence (Fig. 4G). To confirm this is not a non-specific effect of low pHe, levels of G6PDH, a protein unrelated to CRIP2 according to String analysis, were found to be unresponsive to changes in pH (Fig. 4H; see Fig. S7 for quantification of sarcomeric proteins by densitometry and ELISA). The interplay between CRIP2 and contractile proteins may, in part, relate to the binding properties of CRIP2, itself an adaptor protein. To test this, NRVMs incubated at pH 7.4 or 6.4 were collected for an immunoprecipitation experiment using a CRIP2 antibody (Fig. 4I). The immunoprecipitate was analysed by SDS-PAGE followed by silver staining to show bands corresponding to CRIP2 binding partners. A negative control used lysates prepared without the CRIP2 antibody (Fig. 4J). Label-free tandem mass spectrometry analysis of in-gel digested sections was performed to identify proteins. Data filtered with a false discovery rate of 1% identified > 60 peptides, including contractile proteins (Fig. 4K). This finding confirms an association between CRIP2 and contractile proteins, including cTnT, ssTnI, cTnI and MHC α/β, and may explain, in part, why the levels of these proteins change in a coordinated manner in response to pH changes.

### pH-sensitivity of p300/CBP acetylase activity contributes to pH-sensitive gene expression

**Fig. 5:**
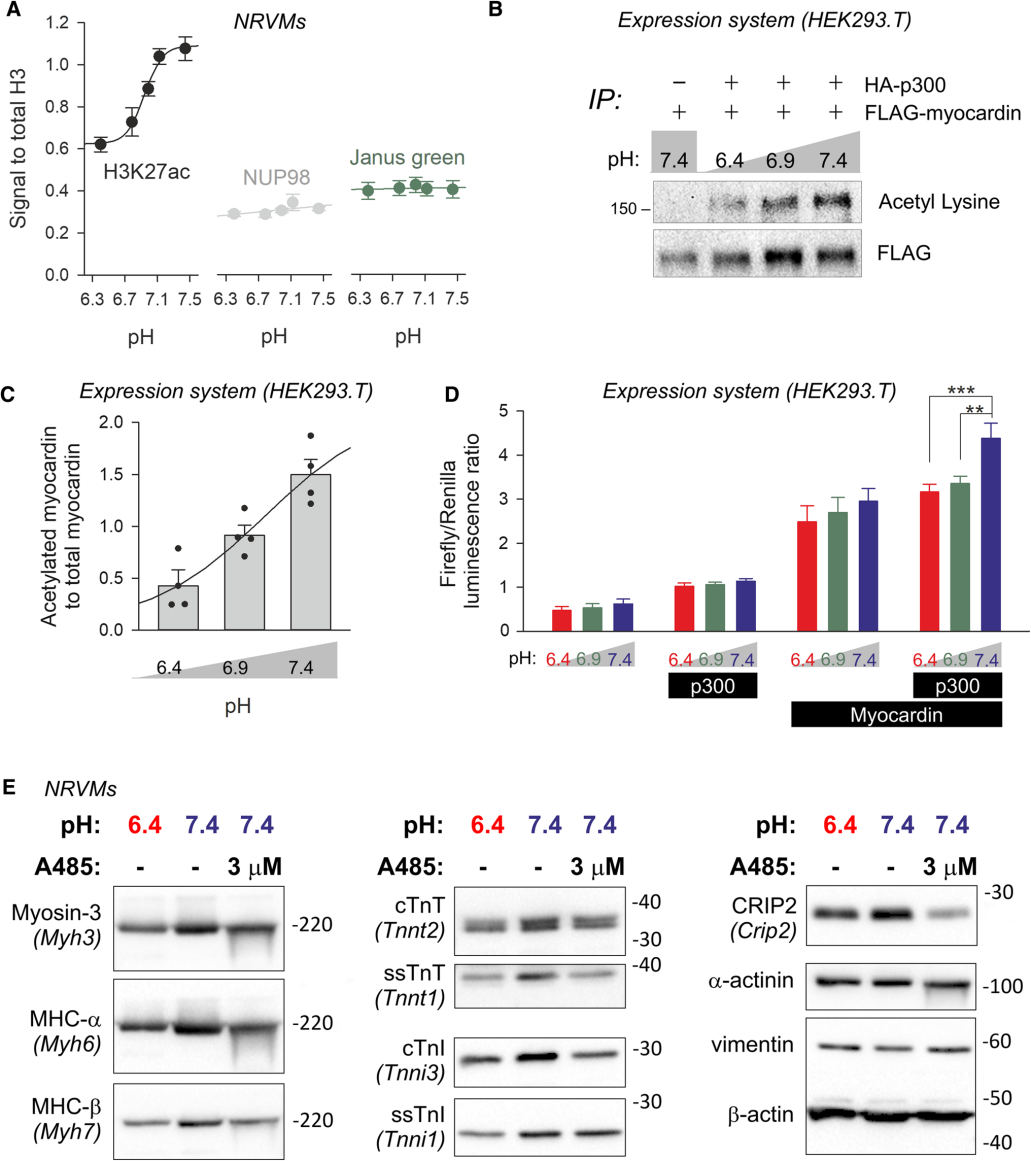
A mechanism of pH-responsive cardiac gene expression involves the pH-sensitivity of p300/CBP. **A** 48 h of incubation of NRVMs in low pH reduces the H3K27ac mark, a readout of p300 activity (5 repeats; significant linear effect of pH; *p* < 0.01). The normalizing control was Janus green and NUP98 (Nucleoporin 98 And 96 Precursor). **B** Acetylation state of immunoprecipitated myocardin co-expressed in HEK293T cells with HA-p300. Final 4 h of culture was performed at pH 6.4, 6.9 or 7.4 by varying [HCO_3_^−^] at constant CO_2_. **C** Quantification of the reduction of p300-dependent myocardin acetylation (4 repeats; significant linear trend by pH; *P* < 0.0007 by mixed-effects modelling regression analysis). **D** Firefly-to-Renilla luminescence ratio measured in HEK293T cells expressing myocardin and p300 (as indicated) with a Renilla reporter and firefly reporter of *Tnnt2*. To assess acute effects of pH, the final 4 h of incubation was at pH 6.4, 6.9 or 7.4. Alkaline pH had a significant stimulatory effect on transcriptional activity when p300 and myocardin were co-expressed (4 repeats; *P < 0.05, **P < 0.01 by two-way ANOVA; significant effect of pH). (E.) Western blots for pH-sensitive myosin heavy chain-family proteins (myosin heavy chain α and β, myosin-3), troponin isoforms (ss-slow skeletal, c-cardiac), CRIP2 and loading control (β actin). β-actin was re-developed using the same membrane as that used for cTnI. α-actinin and vimentin were used as a cardiomyocyte and fibroblast marker, respectively, to determine that the culture had not been enriched in fibroblasts as a result of treatment. Vimentin was re-developed using the same membrane as that used for Myh6. Lysates were prepared after 48 h of incubation at pH 6.4 or 7.4 in the presence or absence of p300 inhibitor A485 (3 µM). Repeats 2 and 3 are shown in Fig. S3, and densitometric quantification is given in Fig. S5

Changes in pHe may trigger an intracellular signalling response by acting on three types of proton-sensing G protein-coupled receptors: GPR4, GPR65 and GPR68 [63]. However, the transcript levels of the corresponding genes measured in NRVMs were low (in the 6^th^ decile when ranked by abundance, equal to less than 3% of average transcript signal) [73]; moreover, their activation typically requires very substantial acidification, below 6.0, which is outside the physiological range and beyond the scope of pH conditions tested herein [29]. We reasoned that the robust response of pH-DEGs relates to more direct actions of H^+^ ions on nuclear regulators, such as histones, transcription factors or their co-activators. The acetylase p300/CBP plays a crucial role in regulating gene expression through actions on the transcriptional co-activator myocardin [80], which must be acetylated [10] in order to activate serum response factor (SRF) [81]. Additionally, p300/CBP plays a major role in acetylating histones at various lysine residues. The importance of p300/CBP in orchestrating the pH-dependence of cardiac gene expression is supported by the observation that a readout [61] of its activity, H3K27ac, is decreased in NRVMs at low pH (Fig. 5A), relative to normalizing controls (Janus green and NUP98). pH-sensitivity of p300/CBP has been predicted [22] and demonstrated in lysates [62], but not evaluated in an intact cellular system. To test for pH-sensitivity of p300/CBP in cells, FLAG-tagged myocardin and HA-p300 were overexpressed in 293 T cells. 24 h after transfection, media were replaced to change pH to 6.4, 6.9 or 7.4 for a further 4 h, during which the acetylation state of myocardin would be expected to change in response to p300 inhibition. FLAG-myocardin was immunoprecipitated with anti-FLAG antibodies and its acetylation state was determined by anti-acetyl lysine antibody by western blot. Myocardin acetylation was found to require p300, and its post-translational modification was determined to be pH-sensitive (Fig. 5B, C; Fig. S12). An alkaline pH is, therefore, necessary for myocardin acetylation by p300/CBP. To test if the pH-sensitivity of myocardin acetylation translates into a change in the expression of genes coding for contractile elements, 293 T cells were co-transfected with myocardin, p300, Renilla luciferase and a Firefly luciferase reporter for *Tnnt2*, a cardiac-specific gene that manifests pH-dependent expression at protein level. Renilla luminescence was used as a normalizing control. Previously, *Tnnt2* was predicted, but not yet shown, to be under myocardin control [50]. Here, we verified that *Tnnt2* expression, quantified as Firefly/Renilla ratio, is significantly increased by myocardin (Fig. 5D). To test for pH-dependence, medium pH was changed for the final 4 h of culture to 6.4, 6.9 or 7.4. The synergistic effect of p300 expression on *Tnnt2* expression was most apparent at alkaline pH (Fig. 5D). Thus, the pH-sensitivity of p300 activity can affect the expression of genes coding for contractile elements.

If p300/CBP inhibition at low pH were linked to the reduction in expression of *Crip2* and ‘contractile’ genes, then its selective pharmacological inhibition at normal pH would be expected to produce a similar downregulation. This was tested in paired experiments where cells were incubated at pH 7.4, pH 6.4 or at pH 7.4 in the presence of A485 (3 µM), a selective p300 blocker. Pharmacological p300 inhibition produced a decrease in CRIP2, MHC α/β, ssTnT, cTnI, similar to the effect of low pH (Fig. 5E; Figs. S6, S7). These findings support the model in which acid-inhibition of p300/CBP acetylase activity downregulates genes coding for components of the contractile apparatus.

**Fig. 6:**
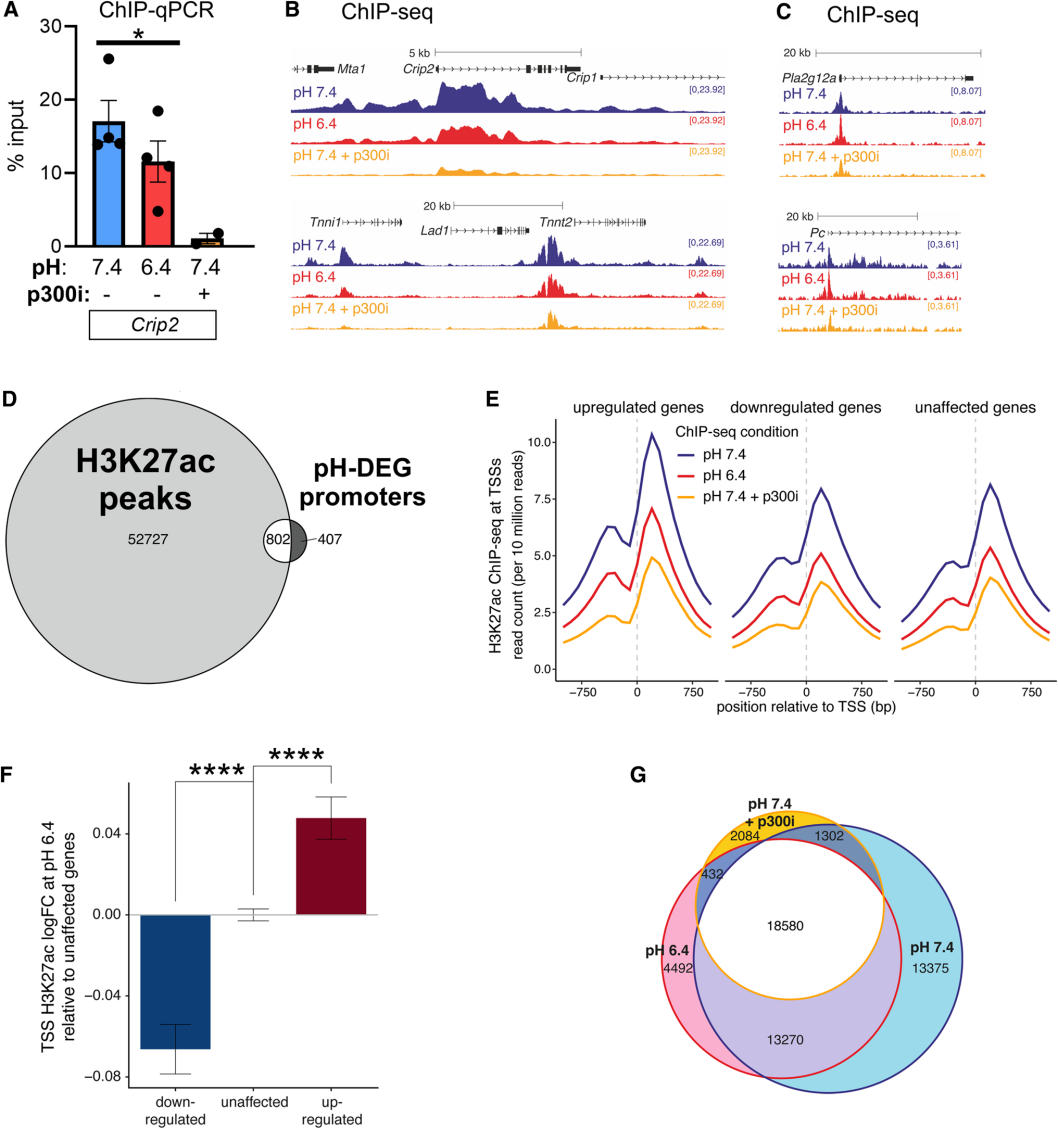
Acidic conditions disrupt H3K27ac at promoters of genes including those coding for contractile proteins.* A* ChIP-qPCR for H3K27ac at the promoter region of *Crip2*. Measurements from NRVMs after 48 h of incubation at pH 6.4 or 7.4 in the presence or absence of p300 inhibitor A485 (3 µM). Mean ± SEM of 5 biological repeats. **B** ChIP-seq tracks for H3K27ac at the indicated genomic loci for down-regulated pH-DEGs (*Crip2* and *Tnni1*) and **C** up-regulated pH-DEGs (*Pla2g12a* and *Pc*). **D** ChIP-seq analysis of NRVMs identified the presence of H3K27ac peaks at promoter regions of the majority (two-thirds) of pH-DEGs. **E** Metaplot demonstrating the mean distribution of H3K27ac across the transcriptional start site (TSS) of genes grouped according to their transcriptional response to acidosis: downregulation, upregulation, or unaffected. Each line represents the mean H3K27ac level under the indicated condition. **F** Difference in mean log fold-change in H3K27ac levels at TSS' of genes that are downregulated or upregulated at low pH, relative to unaffected genes. Mann–Whitney test, ****P < 0.0001. **G** Overlap of H3K27ac peaks identified from ChIP-seq in NRVMs treated as indicated

Having demonstrated that low pH results in a global reduction of H3K27ac (Fig. 5A), we tested whether there was a selective reduction at downregulated genes. ChIP-qPCR measurements quantified the enrichment H3K27ac at the *Crip2* promoter in myocytes incubated under control conditions (pH 7.4), under acidic conditions (pH 6.4) or in the presence of A485 (3 µM) for 48 h (Fig. 6A). Acidic conditions reduced H3K27ac at *Crip2*, albeit to a smaller degree than pharmacological inhibition of p300. This analysis was extended by performing reference-normalized H3K27ac ChIP-seq of pooled NRVM samples. The results confirmed the decrease in H3K27 acetylation at various promoters, including *Crip2* and *Tnni1* at low pH, as well as in the presence of p300 inhibitor (Fig. 6B). Genes, such as *Pla2g12a* or *Pc*, that were up-regulated at low pH did not show a decrease in H3K27ac levels at their promoters, indicating that the effect of pH on histone marks can be gene-specific (Fig. 6C). Two-thirds of pH-DEGs overlapped with H3K27ac peaks at their corresponding promoters (Fig. 6D). Notably, low pH reduced H3K27ac levels at the promoters of genes that were up- or downregulated, consistent with a global inhibition of p300/CBP activity (Fig. 6E), but the reduction in H3K27ac levels was more profound at downregulated gene promoters (Figs. 6F, S13). Upregulated genes, in contrast, had higher levels of H3K27ac at both pH 7.4 and pH 6.4 (Fig. 6E), suggesting that their elevated acetylation may be associated with greater transcriptional resistance to acidosis.

Peak-calling analysis of ChIP-seq data for treatment in pH 7.4, pH 6.4, or 7.4 with p300 inhibition identified 53,535 H3K27ac peaks present in at least one experimental group, with the largest number (46,527; 87%) observed at pH 7.4 (Fig. 6G). The number of H3K27ac peaks was decreased in acidic conditions (38,076; 71%), consistent with an effect of moderate p300 inhibition. Pharmacological p300 inhibition decreases the peak count further (22,398 peaks; 42%). Crucially, most of the peaks lost at pH 6.4 were also absent under p300 inhibitor treatment. Thus, the majority of H3K27ac peaks that require alkaline conditions (i.e. are absent at pH 6.4) can be linked to p300 dysinhibition.

### Transmission of extracellular pH signals into the nucleus is partially regulated

Many of the regulatory elements underpinning pH-responsive gene expression, including p300 and myocardin, reside in the nucleus. The pH that is sensed by these elements is most accurately described in terms of nuclear pH (pHn), which may have a complex relationship with pH in the extracellular space, the origin of the acid/base trigger. Whereas the relationship between pHe and cytoplasmic pH (pHc) is known to be regulated by sarcolemmal acid–base transporters, the relationship between pHc and pHn has not been mapped in cardiac myocytes.

To characterise the coupling between pHc and pHn, we used an imaging approach that pairs bulk cytoplasmic measurements of pH with that in the DNA nano-environment. Various DNA-binding dyes were tested in the HCT116 cell line for pH-sensitivity and compatibility for use with the pHc reporter, cSNARF1. In tandem dual-emission ratiometric imaging mode, fluorescence emitted by the DNA-binding dye and cSNARF1 were excited and imaged sequentially to obtain pHn in nuclear regions and pHc in the cytoplasmic region that surrounds the nucleus (width equal to mean nuclear radius). Cells were superfused with high-K^+^ solution containing the H^+^/K^+^ ionophore nigericin to clamp pHc, and hence pHn, to the pH of the superfusate. The pH-dependence of dyes was thus determined by changing superfusate pH. Hoechst-34580 (H34580) was found to be the most pH-sensitive DNA-binding dye, owing to the presence of a protonatable tertiary amine group because the absence of this chemical moiety in H33258 and H33342 renders the dyes substantially less pH-responsive (Fig. S14). In terms of dynamic range and pK_a_, H34580 had comparable properties to cSNARF1, predicting similar signal-to-noise ratios for pHc and pHn. In comparison, fluorescence from SYTO-type dyes was pH-insensitive (Fig. S15).

**Fig. 7:**
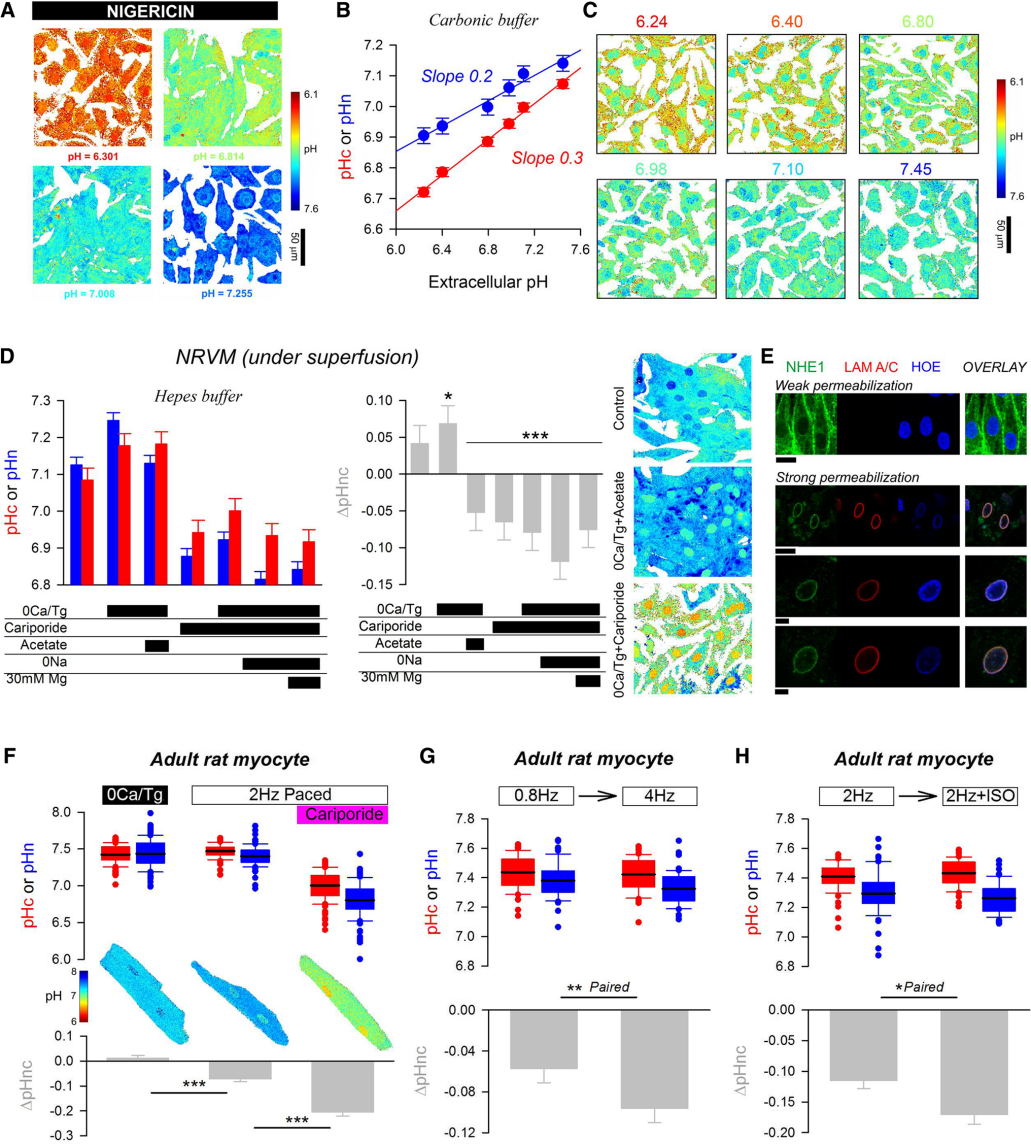
Characterising the relationship between nuclear and cytoplasmic pH. **A** The protonophore nigericin collapses pH gradients across the intracellular compartment of NRVM monolayers, verifying the calibration of nuclear and cytoplasmic dyes. **B** Relationship between pHe, pHc and pHn in NRVMs under incubation conditions following 48 h of treatment in media of pH between 6.24 and 7.44. Each datapoint is mean ± SEM of 583–828 cells/5 isolations. **C** Exemplar images of monolayers showing degree of pHn regulation that becomes more prominent in acidic media. **D** Measurements of pHn/pHc in NRVMs following pharmacological treatments: thapsigargin (10 µM; 10 min) and superfusion with Ca^2+^-free buffer (0Ca/Tg, > 10 min); superfusion with cariporide (30 µM; > 10 min); superfusion with 40 mM acetate (osmotically-compensated; > 10 min), superfusion with Na^+^-free solution (replaced with NMDG) with either 1 or 30 mM Mg^2+^ (> 10 min). Mean ± SEM from 1730 to 2897 cells/4 isolations. (E) Immunofluorescence images of NRVMs stained for NHE1 and lamin A/C, also stained with Hoechst 33,342. Sarcolemmal NHE1 staining, with no lamin A/C signal after weak permeabilization (0.1% Triton X-100, 1 min). Nuclear NHE1 and lamin A/C immunofluorescence after stronger permeabilization (0.5% Triton X-100, 20–30 min) with DNaseI treatment (60 min) to reduce staining artefact due to chromatin. Scale bar is 10 µm. **F** Adult rat ventricular myocytes superfused with Hepes-buffered solution. pHc and pHn measured > 10 min after either Ca^2+^-depletion (0Ca/Tg), 2 Hz pacing, or 2 Hz pacing in presence of 30 µM cariporide. Box plots of > 100 cells/4 hearts (thick line shows mean; error bar span 90th percentile). **G** Effect of increasing pacing frequency and **H** stimulation (> 5 min) with 1 µM isoprenaline (ISO) in paired experiments (> 50 cells/4 isolations) under superfusion with Hepes buffer

Having described the best pH-dye pair in a cell line, the pHn-reporting power of H34580 was tested in myocytes (see Fig. S16 for image processing pipeline). To confirm that H34580 and cSNARF1 report the same value for pH when all intracellular gradients are dissipated, images were taken in the presence of nigericin and high-K^+^ buffer (Fig. 7A). The uniformity of pH signal confirmed that both dyes were calibrated accurately. Next, the pHc/pHn relationship was measured in NRVMs incubated in media over a range of pH, to match the conditions used for RNAseq experiments (Fig. 2A). Under these physiological conditions, both pHc and pHn decreased in more acidic media. However, a difference between pHc and pHn was apparent and became larger in acidic media (Fig. 7B). Whereas pHc decreased by 0.3 units per unit reduction in pHe, pHn was more resilient to change, manifesting as a shallower pHn/pHe slope of 0.2 and the clear emergence of nuclear regions on pH maps at low pHe (Fig. 7C). Thus, an acid signal originating from the extracellular milieu is transmitted to the nucleus, but some degree of pH regulation is exercised at the level of the nucleus.

The positive gradient between pHn and pHc (ΔpHnc = pHn-pHc) persisted and was modestly increased in the absence of physiological CO_2_/HCO_3_^−^ buffer and in the presence of the carbonic anhydrase inhibitor, acetazolamide (Fig. S17A). This finding indicates that HCO_3_^−^-dependent transporters do not maintain nuclear alkalinity. Although the cytoplasm and nucleus are connected via nuclear pores, augmenting H^+^ diffusivity by incubating cells with a high concentration (30 mM) of carnosine (a mobile H^+^-buffer) [70, 71] did not dissipate ΔpHnc (Fig. S17B). This suggests pHn is offset from pHc by an active process that is able to overcome the diffusive leak across nuclear pores, even when supplemented with exogenous mobile buffers. A plausible source of energy for maintaining the pHn/pHc gradient may relate to compartmentalized Ca^2+^ signalling, which is different between the cytoplasm and the nucleus [84]. Nuclear Ca^2+^ signals in NRVMs can be modulated by IP_3_-mobilizing agonists [6, 31, 84], such as endothelin-1 (ET1) or phenylephrine (PE). These agonists were applied for 48 h, followed by pH imaging in agonist-free media. ET1 (100 nM) reduced ΔpHnc, in contrast to a weaker effect of PE (10 µM). As a control, isoprenaline (ISO; 1 µM), an agonist that does not evoke IP_3_ signalling, had no effect on ΔpHnc (Fig. S17C). These observations demonstrate a means of regulating ΔpHnc.

To gain insight into the ionic transport processes that influence pHn, NRVMs were subject to pharmacological interrogation under superfusion with Hepes-buffered solution (Fig. 7D). ΔpHnc became more positive after emptying Ca^2+^ stores with the SERCA inhibitor thapsigargin (Tg, 10 µM) followed by superfusion with Ca^2+^-free buffers (EGTA) to run-down cellular Ca^2+^ stores. Thus, the activity of SERCA acidifies the nucleus, relative to the surrounding cytoplasm. Intriguingly, ΔpHnc flipped polarity (to negative) when the protocol was followed by 10 min exposure to 40 mM acetate, a manoeuvre that activates sarcolemmal Na^+^/H^+^ exchanger-1 (NHE1) and drives Na^+^ influx into the myocyte. Thus, the nucleus-cytoplasm pH gradient may be related to [Na^+^], implicating a Na^+^-dependent process. ΔpHnc also became negative in cells treated with the Na^+^/H^+^ exchanger-1 (NHE1) inhibitor, cariporide (30 µM) for > 10 min, arguing for a role for NHE1. Inhibition of NHE1 protein expressed at the sarcolemma would be expected to reduce both pHc and pHn in tandem, but without changing ΔpHnc polarity; thus the inversion of ΔpHnc suggests a non-sarcolemmal target of cariporide, possibly NHE1 at the nuclear envelope (NE). Previous studies documented evidence for NHE1 in the nucleus [4, 32], and this was confirmed in NRVMs by immunofluorescence of cells subject to a strong permeabilization protocol (Figs. 7E, S18). As a control, milder permeabilization revealed sarcolemmal NHE1 staining only. Taken together, our results suggest that nuclear NHE1 activity may alkalinize the nucleoplasm, an effect that can be blocked pharmacologically and by changing the thermodynamic driving force in Na^+^-overloaded myocytes. Fixed charges held on proteins or DNA in the nucleus may also influence pHn, and to test for this, Ca^2+^-depleted NRVMs were superfused with Na^+^-free solution containing 30 mM Mg^2+^ to import divalents by sarcolemmal Na^+^/Mg^2+^ exchange [1]. This manoeuvre did not change pHn, which suggests that fixed charges do not meaningfully contribute to the difference between nuclear and cytoplasmic pH.

The NHE1-dependent mechanism of nuclear alkalinization was tested in adult rat ventricular myocytes (ARVMs) under superfusion with Hepes-buffered solution. In myocytes with pharmacologically blocked SERCA activity (thapsigargin) and emptied SR Ca^2+^ stores, pHn approaches pHc. When SERCA is activated in electrically paced myocytes (2 Hz), the nucleus acidifies, producing a negative ΔpHnc of − 0.1 units (Fig. 7F). 20-min treatment with cariporide followed by electrical pacing made ΔpHnc more negative (− 0.2 units), suggesting that NHE1 activity normally works against the nucleus-acidifying influence of SERCA. The direction of these pharmacological responses is consistent with results from NRVMs. The link between SERCA activity and pHn can be explained in terms of Ca^2+^/H^+^ exchange mediated by this pump [17, 85] at the inner NE [35]. ΔpHnc became more negative when SERCA-mediated Ca^2+^/H^+^ exchange was stimulated by a higher pacing frequency or stimulation with 1 µM isoprenaline (Fig. 7G, H). Higher pacing frequency or greater Ca^2+^ release increase Ca^2+^ handling fluxes across the sarcolemma and SR membrane, but the only step in this cascade that is stoichiometrically coupled to H^+^ ions is SERCA. For an effect that changes the pHn-pHc gradient, the relevant SERCA pumps must be at the interface, i.e. at the nuclear envelope. In summary, our findings suggest that pHn is set by nuclear SERCA and NHE1 acting against one another.

### Coupling between nuclear and cytoplasmic pH is remodelled in cardiac disease models

**Fig. 8:**
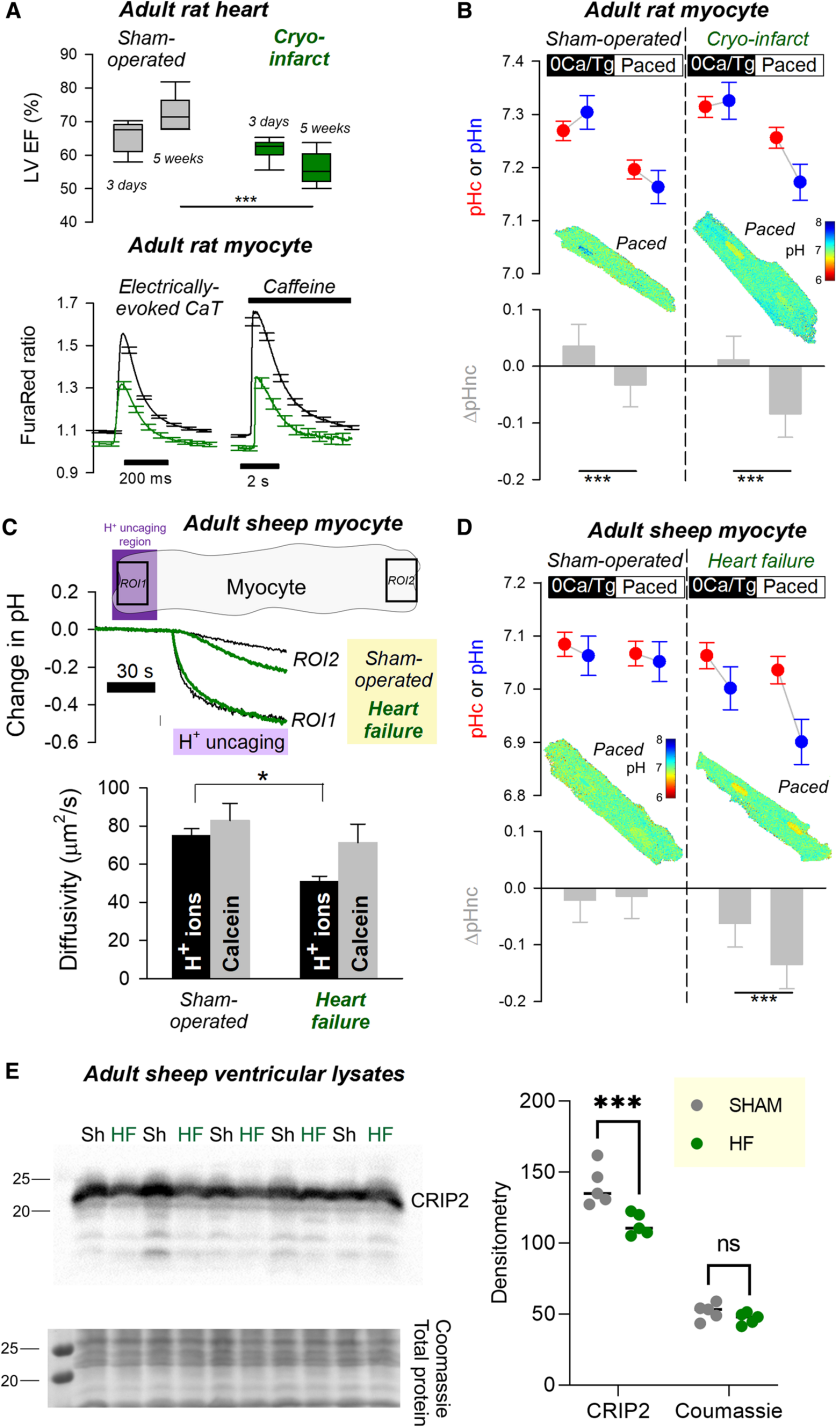
Remodelling of nuclear pH control in models of heart disease.* A* Left ventricular (LV) ejection fraction (EF) measured by cine-MRI at 3 days and 5 weeks after surgery (sham, *N* = 6 or cryo-injury, *N* = 6) and electrically evoked Ca^2+^ transients and 10 mM caffeine-evoked Ca^2+^ release in FuraRed loaded myocytes isolated at 5 weeks post-surgery (> 50 cells from six isolations each). Amplitude of Ca^2+^ responses was significantly reduced in cryo-infarcted hearts (CaT: 0.302 ± 0.01 v 0.477 ± 0.013; *P* = 0.0002; SR load: 0.354 ± 0.17 v 0.644 ± 0.013; *P* < 0.0001). **B** pHn and pHc in rat myocytes from cryo-injured hearts (or sham controls), imaged under superfusion with carbonic buffer either following Ca^2+^-depletion (0Ca/Tg) or 2 Hz pacing. Mean ± SEM of > 117 cells/6 isolations. Hierarchical testing by two-way ANOVA. Type III analysis table: significant effect of 0Ca^2+^/pacing *P* = 0.002. **C** Photolytic H^+^ uncaging protocol to measure H^+^ diffusivity. Best-fit to data gives H^+^ diffusivity. Calcein diffusivity measured by FRAP protocol. **D** pHn and pHc in sheep myocytes from tachypaced failing hearts or sham controls, imaged under superfusion with carbonic buffer either following Ca^2+^-depletion (0Ca/Tg) or 2 Hz pacing. Mean ± SEM of > 120 cells/5 isolations. Hierarchical testing by two-way ANOVA. Type III analysis table: significant effect of 0Ca^2+^/pacing *P* = 0.002; significant interaction between 0Ca^2+^/pacing and heart (sham/failure) *P* = 0.003. **E** Western blot showing Crip2 downregulation in heart failure lysates compared to sham (Sh) from five sham and five HF animals. Quantification and analysis by two-way ANOVA. Significance: *P* = 0.003

Failing hearts are often associated with dysregulated ionic signalling, which may affect nuclear pH, and then contribute towards altered gene expression. This was tested in myocytes isolated from a cryo-injured rat heart [53] or an ovine tachypaced model of heart failure [9], two in vivo models presenting with a weakened contraction. At 5 weeks after cryo-injury, ejection fraction (EF), the amplitude of electrically evoked Ca^2+^ transients (CaTs) and the size of the sarcoplasmic reticulum (SR) Ca^2+^ store were decreased (Fig. 8A). Electrically paced myocytes isolated from cryo-injured hearts at 5 weeks post-surgery had a notably larger pHn-pHc gradient (Fig. 8B). In the sheep model of heart failure, the major component of pH-related remodelling related to H^+^ diffusion, which was considerably reduced relative to sham-operated hearts (Fig. 8C). This was not due to a non-specific change in diffusive tortuosity, as calcein diffusivity was unaltered. Previously, we showed that pH buffering in the nucleus is mainly supplied by mobile buffer synthetized in the cytoplasm [30]. Thus, the reduction in H^+^ diffusivity implies a decrease in nuclear pH buffering owing to the depletion of diffusible buffers. This situation makes it more likely for pHn to change relative to pHc. Consistent with this, the nuclei of paced myocytes isolated from sheep failing hearts were considerably more acidic than sham-controls (Fig. 8D). As expected from lower pHn, levels of CRIP2 were reduced in tachypaced hearts, compared to sham-controls (Fig. 8E). Although the mechanism underpinning lower Crip2 expression in heart failure may be multi-factorial, it may be explained by the pH-sensitivity of CRIP2 and may also indicate that pHn is the more direct trigger, compared to pHc.

## Discussion

**Fig. 9:**
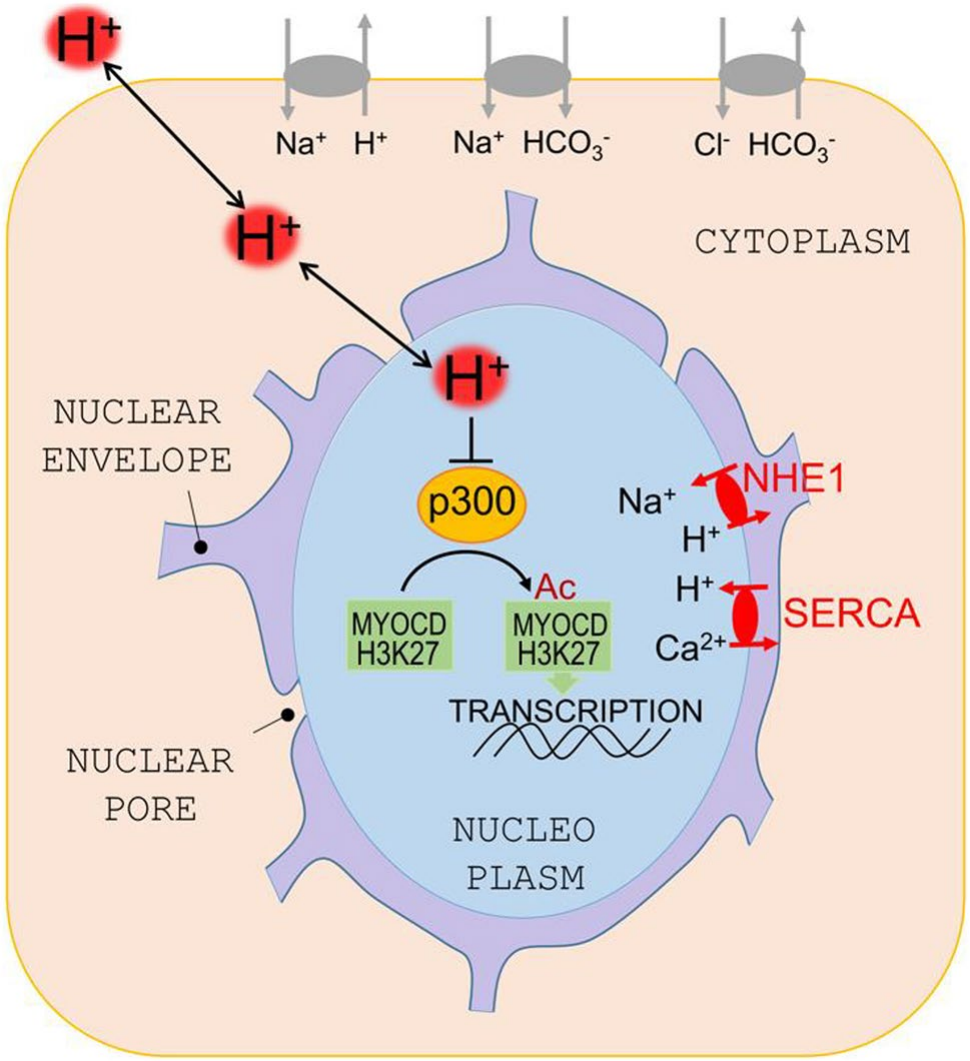
Schematic for a proposed model by which pH influences cardiac gene expression

This study presents evidence for pHe non-uniformity in the postnatal heart, which can meaningfully influence the expression of a subset of cardiac genes, notably those encoding contractile components. We also show how an acid signal, originating from the extracellular space, is transmitted to the nucleoplasm, where it can modulate pH-sensitive nuclear regulators. Among these regulators is p300/CBP, which we find to have reduced acetylase activity at low pH. The acid-evoked decrease in acetylation of histones, myocardin and possibly other proteins is a mechanism by which H^+^ ions regulate gene expression (Fig. 9). We also propose an ionic mechanism regulating the coupling between cytoplasmic and nuclear pH, which itself can be altered in disease.

Based on the distribution of pHLIP, we described acidic niches in the inner myocardial wall (IMW) of early postnatal hearts, which dissipate in the adult. The likely cause of this pH non-uniformity is spatial variation in capillary-dependent washout of the acidic end-products of metabolism. Consistent with this, coronary vasculature is formed later in the IMW, compared to other regions of the heart, and remains incomplete even at postnatal day-7 [72]. As vascular perfusion matures, pHe measured across the myocardium becomes more uniform and remains as such in the healthy adult heart. Thus, early development provides a physiological context for pH to influence gene expression at a critical developmental milestone and is likely to work alongside the actions of hypoxia.

In order for pH to be a meaningful modulator of cardiac gene expression, it would need to strategically target a subset of genes relevant to heart function. Overall, pH directly influenced the expression of only 7% of cardiac genes, which is a striking degree of specificity for an otherwise broad-spectrum modulator of heart physiology. Of the ~ 1200 pH-DEGs identified by transcriptomics, only 54 manifested a sufficiently strong pH-response when measured in terms of protein abundance by unbiased proteomics. Indeed, manual validation of selected pH-DEGs by immunoreactivity assays did not consistently show the expected pH-dependence at the protein level. Thus, the number of genes directly regulated by pH at expression level is modest, which gives the pH signal a good degree of selectivity, as required by a *bona fide* gene regulatory cue.

In terms of biological processes, genes belonging to the “striated muscle contraction” pathway showed the most significant pH-dependence. At protein level, pH-sensitivity was confirmed for troponins and myosin heavy–chain isoforms. Postnatal development involves major changes in troponin isoform expression, highlighting these as the finely regulated element of contraction. Our findings add pH-sensitivity to the list of their modulators. A physiological context for exercising the pH-sensitivity of “contractile” genes may relate to the postnatal maturation of vasculature in the myocardium. As blood perfusion improves, genes coding for contractile elements become disinhibited, which matches the more conducive ionic milieu for contraction. Indeed, early neonatal life is a period associated with profound changes in troponin isoform expression [58, 59], which may, in part, be driven by their pH-sensitivity.

Among the genes with the highest abundance of transcript, *Crip2* showed a striking pH-dependence of expression at gene and protein level. Although little is known about the biological role of this gene in the heart, it is a putative transcription factor [15, 69, 89] with two LIM zinc-binding domains [40, 76] that may colocalize with troponin T [82] and other sarcomeric proteins [74]. Herein, we confirm that various components of the contractile apparatus, including cTnT, ssTnI, cTnI and MHC α/β, are CRIP2 binding partners, which may in part explain why their protein levels change in a coordinated manner during changes in pH. The diverse functions of CRIP2 may explain why this protein is detected in both the cytoplasm and nucleus. *Crip2* has been implicated in cardiac development through actions on chromatin remodelling [12], and its deletion results in ultrastructural changes and abnormal hypertrophy [64]. Based on our observations, *Crip2* expression may be a useful marker of myocardial acidity. Indeed, a reduction in *Crip2* levels has been described in patients with myocardial ischaemia [88], and we find a similar downregulation in the failing sheep hearts that also develop profoundly acidic nuclei.

Our study implemented a tandem ratiometric imaging method to characterise how a change in extracellular pH affects cytoplasmic and nuclear pH. This was used to address the question of how acid signals gain access to cardiac genes. Hoechst 34,580 was found to yield the most accurate and direct readout of pH at the interface between DNA and nuclear proteins (e.g. histones, transcription factors). The pH in this nanodomain will not necessarily match that of bulk nucleoplasm, and thus gradients (ΔpHnc) may have gone unnoticed in previous studies. Whilst we confirm that acid signals arising in the myocardial interstitium are able to gain access to the nucleus, our findings show that the coupling between these compartments can be regulated. The cardiac nucleus can exercise a degree of pH autonomy, manifesting as a gradient, ΔpHnc, that spans a range of 0.2 units and is the largest example of a stable pH gradient in myocytes, other than those across a contiguous membrane. Cariporide-sensitive NHE1 activity makes nucleoplasm more alkaline, whereas Ca^2+^/H^+^ exchange has the opposite effect. Nuclear NHE1 immunoreactivity has been described previously by others [4, 32] and confirmed herein. Further studies are needed to test our model using gene ablation and to then link pHn regulation with gene expression responses. Strikingly, nuclei were more acidic in myocytes from disease models presenting with weakened contractions (tachypacing [8, 38] and cryo-injury [19, 39]). Since pHn is the direct modulator of genes, this observation may indicate a role for pH-responsive genes in the pathological changes and merits further investigation.

There may be a myriad of mechanisms linking pH to gene expression, and a limitation of our study is that we described only one such mechanism, involving p300/CBP. A decrease in pHn exerts an inhibitory effect on the acetylase activity of p300, which results in a decrease in H3K27 acetylation as well as a decrease in the acetylation of myocardin, a co-regulator of serum response factor (SRF). Myocardin de-acetylation reduces its transcriptional activity for *Tnnt2*, an exemplar gene of the cardiac contraction pathway. Given that CRIP2 is another transcriptional co-activator of SRF in a complex with p300 [12, 13], the pH-dependence of *Crip2* expression would synergise with acid-inhibition of p300 catalysis to favour cardiac muscle gene expression at high pHn. Furthermore, the *Crip2* promoter has been shown to contain SRF-binding sites [13, 14, 46]. Low pH was found to decrease H3K27ac levels at promoters of genes, including those coding for contractile elements. This effect was replicated by p300 inhibition at pH 7.4 and, furthermore, most of the acid-ablated H3K27ac peaks also responded to pharmacological p300 inhibition.

In conclusion, we propose that acid–base chemistry, a gauge of metabolic-perfusion status, can influence the expression of a subset of genes relevant to cardiac function. A physiological context for this pH-sensitivity is in the developing heart, where an alkaline signal upregulates the expression of contractile proteins. We interpret this to be a mechanism for ensuring that the contractile apparatus becomes fully assembled once there is adequate perfusion to support the necessary metabolic rate. Any myocardial acidification that develops in later life, for example such as during ischaemia, may result in weakened contraction due to the reversal of this pH-operated process. A future direction for this line of work is to investigate whether interventions that raise interstitial pH, such as oral bicarbonate buffer therapy [47], could strengthen contraction of the under-perfused, failing heart. It would also be important to understand the contribution of acidosis towards changes in gene expression in cardiac diseases involving an element of ischemia. These studies could also critically evaluate the diagnostic value of pH-responsive genes, such as CRIP2.

## Supplementary Information

Below is the link to the electronic supplementary material.

Supplementary file1 (DOCX 8851 KB)

Supplementary file2 (CSV 397 KB)

Supplementary file3 (CSV 322 KB)

Supplementary file4 (CSV 2 KB)

## Data Availability

All data are made available in the supplement or upon request to the corresponding author. Proteomics datasets are available to download. RNA-seq and ChIP-seq data generated in this study have been deposited in the Gene Expression Omnibus (GEO) with the accession code GSE196439.
