# Supplementary material for: Alkaline nucleoplasm facilitates contractile gene expression in the mammalian heart
Source: Basic Res Cardiol. 2022 Mar 31;117(1):17. doi: 10.1007/s00395-022-00924-9 (PMC8971196; doi:10.1007/s00395-022-00924-9)
Supplement: Supplementary file 1 — Supplementary file1 (DOCX 8851 KB) [file 395_2022_924_MOESM1_ESM.docx]

**GLOSSARY**

pHn Nucleoplasmic pH

pHc Cytoplasmic pH

ΔpHnc Gradient (difference) between pHn and pHc

pHLIP pH-(low)-insertion-peptide

NHE1 Na^+^/H^+^ exchanger 1 (SLC9A1)

SERCA Sarco/endoplasmic reticulum Ca2+-ATPase

P1/P7/P21 Postnatal day 1/7/21

ELISA Enzyme-linked immunosorbent assay

DEG Differentially expressed gene

DAP Differentially abundant protein/peptide

TF Transcription factor

KEGG Kyoto Encyclopedia of Genes and Genome

NRVM Neonatal rat ventricular myocyte

AVM Adult ventricular myocyte

EF Ejection fraction

HF Heart failure

**SUPPLEMENTARY METHODS**

***HCT116 cells.*** HCT116 cells were purchased from ATCC. Cells were cultivated in DMEM (Life technologies, Cat. No. 41965-039) supplemented with 10% FBS (Sigma-Aldrich) and 1% PS (100 U/mL penicillin, 100 µg/mL streptomycin; Sigma-Aldrich). For microscopy, cells were plated and superfused using 4-well Ibidi chambers.

***Details of preparation of cytoplasmic (soluble) and residual (nuclear) fractions***. PER™ Nuclear and Cytoplasmic Extraction Reagents (Pierce, ThermoFisherScientific) were used for fractionating. Briefly, NRVM cells were washed twice with ice cold PBS, trypsinized, resuspended in PBS and pelleted for 3 minutes at 1000 rpm. Next, cellular pellet was resuspended in 200µl of ice-cold CERI, vortexed and lysed for 10 minutes on ice. Then 11 µl of ice-cold CERII was added to the lysate, vortexed and incubated for additional 1 minute. CERI+II lysate was then centrifuged at 18000 g for 5 minutes at 4°C and supernatant (cytoplasmic/soluble fraction) was transferred to clean tube. Pellet was resuspended in 130µl of ice-cold NER solution which was followed by four cycles of 15s vortexing and 10-minute incubation on ice. At the end, the NER sample was centrifuged at 30,000 g for 10 minutes at 4°C and supernatant (residual/nuclear fraction) was transferred to a clean tube. Both fractions were stored at -80°C until they were needed for experiments. Manufacturer’s protocol was followed, using a CERI:CERII:NER ratio of 200 µl:11 µl:130 µl to allow enough sample for estimating protein content in residual/nuclear fraction and for western blot analysis of samples intended for proteomics and the proteomic analyses.

***Sample loading and positioning in polyacrylamide gels.*** Briefly, samples were divided into four equal aliquots and loaded across two gels, such that each gel had a duplicate of each sample, separated by a protein marker. After protein separation, both gels were transferred onto separate PVDF membranes, and cut vertically to separate the duplicate lanes on either side of the protein marker. This produced four membranes that had been loaded in the same manner. Next, the membranes were cut horizontally so that proteins of high and low molecular weight could be developed from the same membrane. The top sections of membranes were used to develop myosin isoforms and alpha-actinin, whereas the bottom sections would be used to develop Crip2, troponins and beta-actin (as the loading control).

***pHLIP synthesis.*** pHLIP peptide Var3 was synthesized and purified by CS Bio Co. pHLIP peptide and Cy5.5-maleimide (Lumiprobe) were dissolved in DMSO. Peptide concentration was calculated by absorbance at 280 nm in methanol, using extinction coefficient ε_280_ = 12,660 M^-1^ cm^-1^. Cy5.5-maleimide concentration was calculated by absorbance at 684 nm in methanol, using extinction coefficient ε_800_ = 198,000 M^-1^ cm^-1^. Cy5.5-maleimide was mixed with the peptide at the molar ratio of 1:1. 100 mM sodium phosphate buffer, pH 7.2, containing 150 mM NaCl was saturated with argon and added to the reaction mixture (1/20 of total volume). Reaction mixture was incubated at room temperature for 2 hours and the reaction progress was monitored by the analytical reverse phase HPLC (Zorbax SB-C18 column 4.6 x 250 mm, 5 µm; Agilent Technologies; the gradient of binary solvent system using water and acetonitrile with 0.05% TFA for 20-80% over 30 min). Cy5.5-pHLIP was purified by the reverse phase HPLC (Zorbax SB-C18 columns 9.4 x 250 mm, 5 µm; Agilent Technologies, the same gradient, over 40 min), lyophilized and characterized by SELDI-TOF mass spectrometry.

***Cine Magnetic Resonance Imaging.*** Cardiac structure and function were assessed *in vivo* using cine MRI at 3 days and 5 weeks post-surgery using a 7 T horizontal bore system (Varian Medical Systems, Yarnton, UK), a 72 mm ^1^H volume transmit RF coil and a ^1^H phase array 4-channel RF surface receive coil (RAPID Biomedical GmbH, Germany). Anesthesia was induced at 2.5-3% isoflurane in oxygen and nitrous oxide (4:1, total of 2 L/min) and maintained at 2% isoflurane. Body temperature was maintained using air heating, and a two-lead ECG, placed subcutaneously into the upper forelimbs, was used for cardiac gating. Following global shimming, axes of the heart were defined using a series of sagittal and axial scout ECG-gated fast low angle shot (FLASH) images. Once the short axis orientation was defined, 13-14 contiguous short axis slice images were acquired covering the entire heart. Imaging parameters were as given; field of view 51.2 x 51.2 mm, matrix size: 192 x 192, slice thickness: 1.6 mm, TE/TR: 1.43/4.6 ms, Gaussian RF excitation pulse: ~25 degrees and 4 averages. The freehand drawing function of ImageJ (National Institutes of Health, USA) was used to outline epicardial and endocardial borders. End-diastolic (ED) and end-systolic (ES) frames were selected as those with the largest and smallest cavity volumes respectively, and the maximum dimension was recorded as end systolic and diastolic lumen, respectively. Measurements from all slices were summed to calculate ED volume (EDV), ES volume (ESV), stroke volume (SV = EDV – ESV) and ejection fraction (EF = SV/EDV). In each MRI slice, the length of akinetic epicardium and the total length of epicardial surface in a slice were recorded as akin_i_ and total_i_ for the i-th slice. Then, relative infarct size was defined as sum of piecewise akin/total ratios for all slices.

***Calcium imaging.*** FuraRed (Thermo Fisher Scientific, F3021) was used for ratiometric imaging in dual excitation mode (alternating excitation at 490 nm and 435 nm) with a CoolLED pE-4000 LED light source. Fluorescence (645 ± 37.5 nm) was recorded with a QImaging camera. Cells were loaded with the compounds by AM-loading at room temperature for 10 minutes. To measure electrically evoked CaTs, myocytes were superfused at 37°C with solution containing (in mM): 135 NaCl, 4.5 KCl, 20 HEPES, 1 CaCl_2_, 1 MgCl_2_, and 11 glucose (pH 7.4) at 37°C and paced at 2Hz. To evoke release of Ca^2+^ from the SR, a solution containing 10 mM caffeine was rapidly presented to cells.

***Photolytic H^+^ ion uncaging.*** cSNARF1 loaded NRVM cells were superfused with Hepes buffered solution containing 30 µM DMA (Sigma/Merck) and 0.5 mM 6-nitroveratraldehyde (NVA; Sigma/Merck). Protons were uncaged by 405 nm laser from NVA in square ROI placed at one end of spindle-shaped myocytes. The diffusive spread of H^+^ ions was recorded across the cell using cSNARF1 fluorescence. Imaging and uncaging alternated to produce a near-constant source of acid. The pH time courses were then used to calculate the apparent diffusion coefficient as described previously^1,2^.

***In-cell ELISA.*** NRVMs (60,000 cells/well) were plated into fibronectin (Sigma/Merck) pretreated 96-well adherent tissue plate and cultured by standard protocol. Cells were then washed twice with ice-cold PBS and fixed with cold methanol (-20°C) for 10 minutes at 4°C. This was followed by blocking the cells in 0.3% H_2_O_2_ in PBS solution for 10-15 minutes at RT and in 10% FCS in 0.1%PBS-Tween 20 for at least 1 hr at RT. Blocking was followed by incubation with primary antibodies for 1 hour at RT diluted 1:300 in blocking solution: polyclonal rabbit Abs against H3K27-Ac, mouse anti-total H3 (CST), rabbit polyclonal against NUP98 (CST) and mouse monoclonal anti-GAPDH antibody (Proteintech). Cells were washed 4x with 0.1% PBS-Tween 20 and incubated another 1hr at RT with goat anti-rabbit IgG H&L-HRP polymer (Abcam) or with goat anti-mouse IgG H&L-HRP polymer secondary antibodies (Abcam) in dilution 1:1 in blocking buffer. Cells were then washed again 4x 0.1% PBS-Tween 20, developed with OPD for up to 5 minutes. Absorbance was measured at 490nm using Cytation5 plate reader (Biotek). Measurements were performed on 4 independent isolations. To normalize for cell number, Janus green and NUP98 were used. Janus green (Abcam) is a dye that binds to mitochondrial proteins. Briefly, cells were incubated with Janus Green solution for 10 minutes at RT and washed 5x with ultrapure water. Then 0.1ml of 0.5M HCl was added to each well, followed by 10 min incubation and absorbance measurement at 595nm.

***CRIP2 immunoprecipitation and label-free mass spectrometry of in-gel digested samples.*** 5.10^6^ NRVM cells were plated onto 10cm fibronectin coated TC Petri dishes. After 48hr incubation at pHe 6.40 (acidic) or 7.44 (alkaline), cells were washed twice with ice-cold PBS, lysed in RIPA (CST) containing Halt inhibitors of proteases and phosphatases (ThermoFisher Scientific). Lysates were centrifuged at 18000 rpm for 20 min at 4°C. Supernatant was then used for estimation of protein content using BCA assay (Pierce, ThermoFisher Scientific). 0.468 mg of total protein per pH condition was used for immunoprecipitation with (experimental group) or without (negative control) anti-CRIP2 antibody. Briefly, each lysate was first pre-cleared with 60μl of Protein A/G Magnetic Agarose Beads (Pierce, ThermoFisher Scientific) at 4°C for 3-5 hrs. Then, each precleared lysate was split to half and transferred to a clean tube with 50μl of fresh Protein A/G Magnetic Agarose Beads. Anti-CRP-2 rabbit polyclonal antibody (ab151496, Abcam) was added to two lysates (6.40 and 7.44) in dilution of 1:100. Another set of lysates (6.40 and 7.44) was incubated with magnetic A/G beads in the absence of antibody, performed as a control for non-specific binding. Lysates were incubated with or without anti-CRIP2 antibody overnight at 4°C. The next day lysates were removed, and magnetic A/G beads were washed 4x with PBS, beads were then resuspended in 1x Laemmli buffer containing β-mercaptoethanol, heated to 65°C for 5 minutes and loaded onto precast 4%-15% polyacrylamide gradient gel (Bio-Rad). After protein separation gels were silver stained or were blotted onto PVDF membrane and CRIP2 was developed using mouse monoclonal antibody against CRIP2 (C-2, Santa Cruz Biotechnology) and anti-mouse goat HRP conjugated secondary antibody (ThermoFisher Scientific). Gels used for mass spectrometry were first silver stained using Pierce Silver Stain for Mass Spectrometry kit (ThermoFisher Scientific) according to manufacturer’s recommendation and 5 gel areas per condition were cut out, transferred to clean tube, chopped to smaller pieces, washed and and transferred into protein Lobind Eppendorf tubes. Destaining and in-gel digestion were performed at the Advanced Proteomics Facility at South Parks Road (University of Oxford). Gel pieces were destained in 100ul of 50% acetonitrile and 50% 100mM ammonium bicarbonate solution for 30 minutes at 37°C shaking at 600rpm. Supernatant was discarded and gel pieces were incubated with 500ul of 100% acetonitrile. Supernatant was discarded and dried gel pieces were resuspended into 100ul of 10mM TCEP (tris(2-carboxyethyl)phosphine) for 30 minutes at RT. Supernatant was discarded and dried gel pieces were resuspended into 50mM 2-CAA (2-Chloroacetamide) for 30 minutes at RT in the dark. Supernatant was discarded and gel pieces were incubated with 500ul of 100% acetonitrile. Dried gel pieces were further resuspended into 50ul of 10ng/ul trypsin solution in 100mM ammonium bicarbonate (digestion buffer) and incubated for 20h at 37°C shaking at 850rpm. After digestion for peptide recovery, the supernatant (SN1) was transferred into a new protein Lobind Eppendorf tube; 100ul of 5% formic acid/acetonitrile (1:2 volume/volume solution) was added to the gel pieces and incubated for 15 minutes at 37°C shaking at 850rpm. Supernatant (SN2) was collected and combined with SN1. Gel pieces were incubated with 100ul of 5% formic acid/acetonitrile (1:2 volume/volume solution) was added to the gel pieces and incubated for 15 minutes at 37°C shaking at 850rpm. Supernatant (SN3) was collected and combined with SN1 and SN2. Recovered peptides were dried in a speed vac and resuspended into 5% acetonitrile, 5% formic acid before LC-MS/MS analysis. Peptides were separated by nano liquid chromatography (Thermo Scientific Easy-nLC 1000) coupled in line a Q Exactive mass spectrometer equipped with an Easy-Spray source (Thermo Fischer Scientific). Peptides were trapped onto a C18 PepMac100 precolumn (300µm i.d.x5mm, 100Å, ThermoFischer Scientific) using Solvent A (0.1% Formic acid, HPLC grade water). The peptides were further separated onto an Easy-Spray RSLC C18 column (75um i.d., 50cm length, Thermo Fischer Scientific) using a 15 minutes linear gradient (15% to 35% solvent B (0.1% formic acid in acetonitrile)) at a flow rate 200nl/min. The raw data were acquired on the mass spectrometer in a data-dependent acquisition mode (DDA). Full-scan MS spectra were acquired in the Orbitrap (Scan range 350-1500m/z, resolution 70,000; AGC target, 3e6, maximum injection time, 50ms). The 5 most intense peaks were selected for higher-energy collision dissociation (HCD) fragmentation at 30% of normalized collision energy. HCD spectra were acquired in the Orbitrap at resolution 17,500, AGC target 5e4, maximum injection time 120ms with fixed mass at 180m/z. Charge exclusion was selected for unassigned and 1+ ions. The dynamic exclusion was set to 5 s. For protein identification, tandem mass spectra were searched using SEQUEST HT within Proteome discoverer PD1.4 (Thermo Fischer Scientific, version 1.4.0.288) against a database containing 30210 protein entries combining protein sequences from *Rattus Novegicus (UP000002464, Uniprot release 2021-11-11)* and common contaminants. During database searches, cysteines (C) were considered to be fully carbamidomethylated (+57,0215, statically added), methionine (M) to be fully oxidised (+15,9949, dynamically added), all N-terminal residues to be acetylated (+42,0106, dynamically added). Two missed cleavages were permitted. Peptide mass tolerance was set at 50ppm and 0.02 Da on the precursor and fragment ions respectively. Protein identification was filtered at FDR below 1%.

**Additional references**

1. Dovmark TH, Hulikova A, Niederer SA, Vaughan-Jones RD, Swietach P. Normoxic cells remotely regulate the acid-base balance of cells at the hypoxic core of connexin-coupled tumor growths. *FASEB J* 2018;**32**:83-96.

2. Swietach P, Spitzer KW, Vaughan-Jones RD. pH-Dependence of extrinsic and intrinsic H(+)-ion mobility in the rat ventricular myocyte, investigated using flash photolysis of a caged-H(+) compound. *Biophys J* 2007;**92**:641-653.

**SUPPLEMENTARY TABLE S1**

**Table S1: RNAseq quality control experiments.** RNA integrity numbers (RINs). Sets 3, 4 and 6 were selected for RNAseq.

|  |  |  |  |  |  |  |
| --- | --- | --- | --- | --- | --- | --- |
| Set 🡪 | **1** | **2** | **3** | **4** | **5** | **6** |
| **24.4 mM NaHCO3** | 10 | N/A | 10 | 10 | 9.9 | 10 |
| **12.2 mM NaHCO3** | 10 | N/A | 9.9 | 10 | 10 | 10 |
| **9.15 mM NaHCO3** | 10 | N/A | 10 | 10 | 10 | 10 |
| **6.1 mM NaHCO3** | 10 | N/A | 10 | 10 | 10 | 10 |
| **3.05 mM NaHCO3** | 10 | N/A | 10 | 10 | 9.9 | 9.9 |


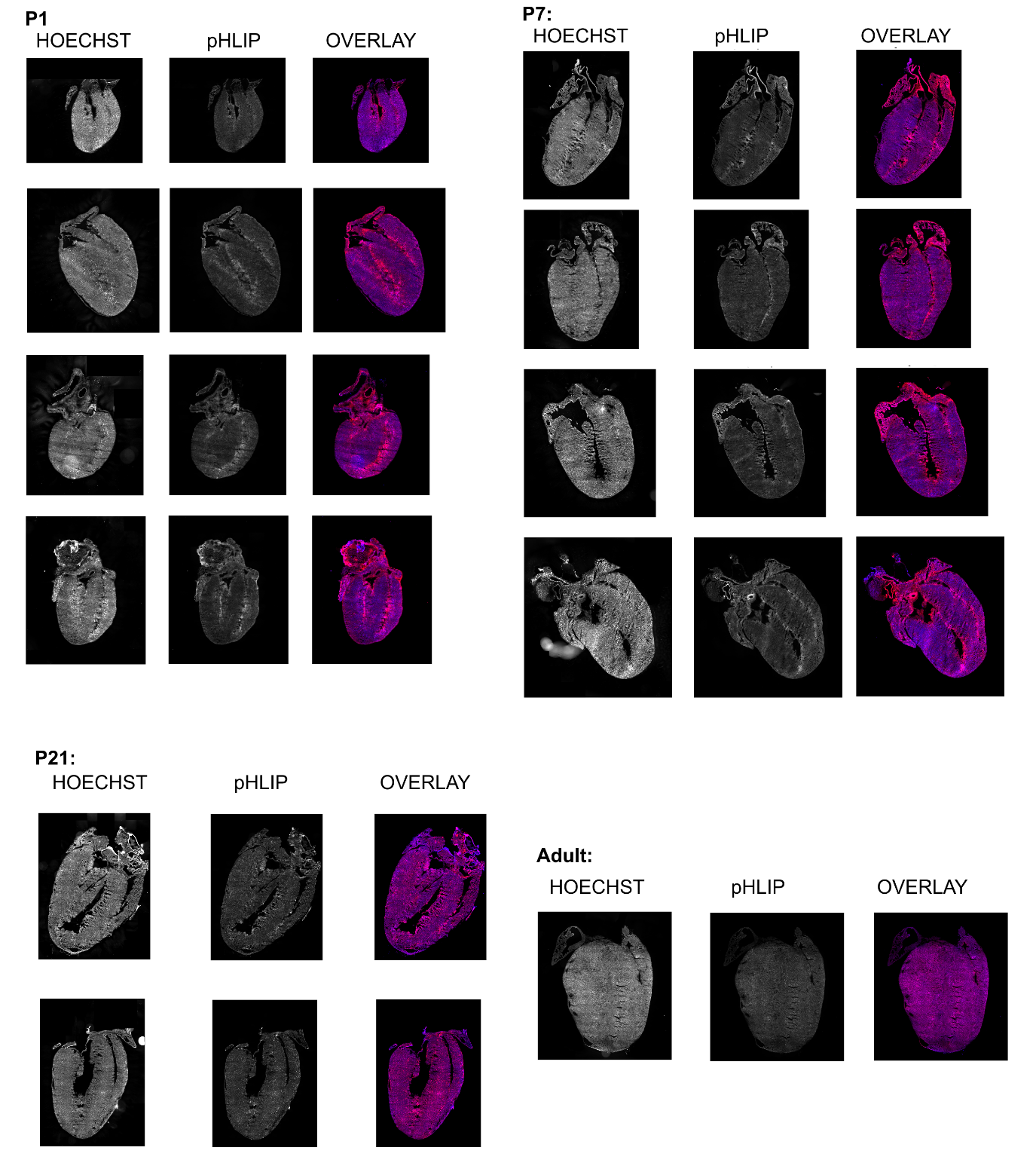


**Figure S1: pHLIP and Hoechst fluorescence for the heart sections shown in Figure 1.** See Figure 1 for scale bars.


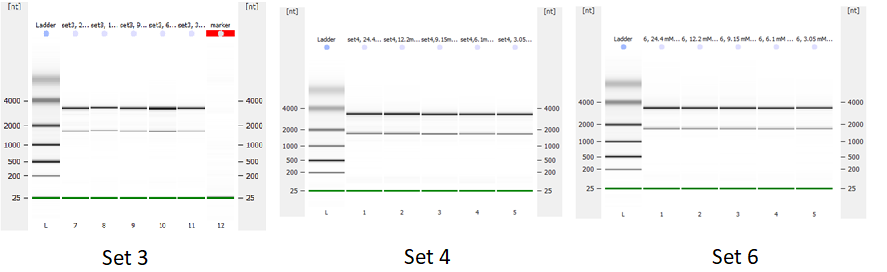


**Figure S2: RNAseq quality control experiments.** 18S and 28S RNA bands for sets 3, 4 and 6 determined by bioanalyzer. The samples were confirmed for RNAseq.

**
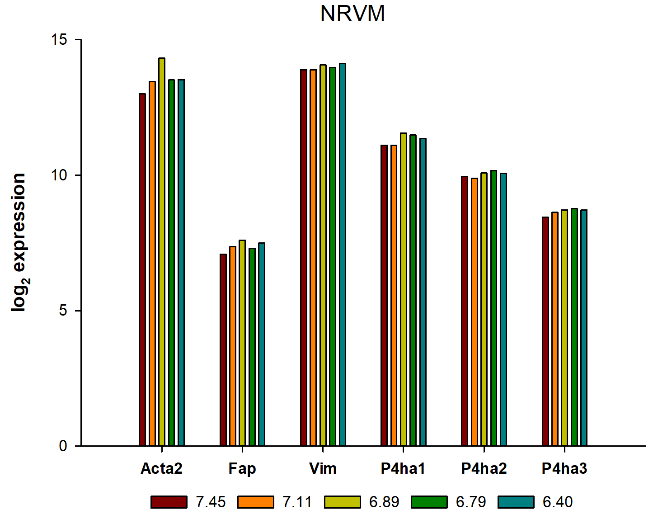
**

**Figure S3: Markers of fibroblasts in samples used for RNAseq.** Fibroblasts are a potential contaminant of NRVM culture, and if their relative abundance changes as a function of pH, at least some DEGs in such co-cultures could be false-hits resulting merely from an enrichment in fibroblasts. To test this, various markers of fibroblasts were analysed in the RNAseq database. These did not change as a function of pH, indicating that the fibroblast population is not altered during pH treatments of NRVMs.

**
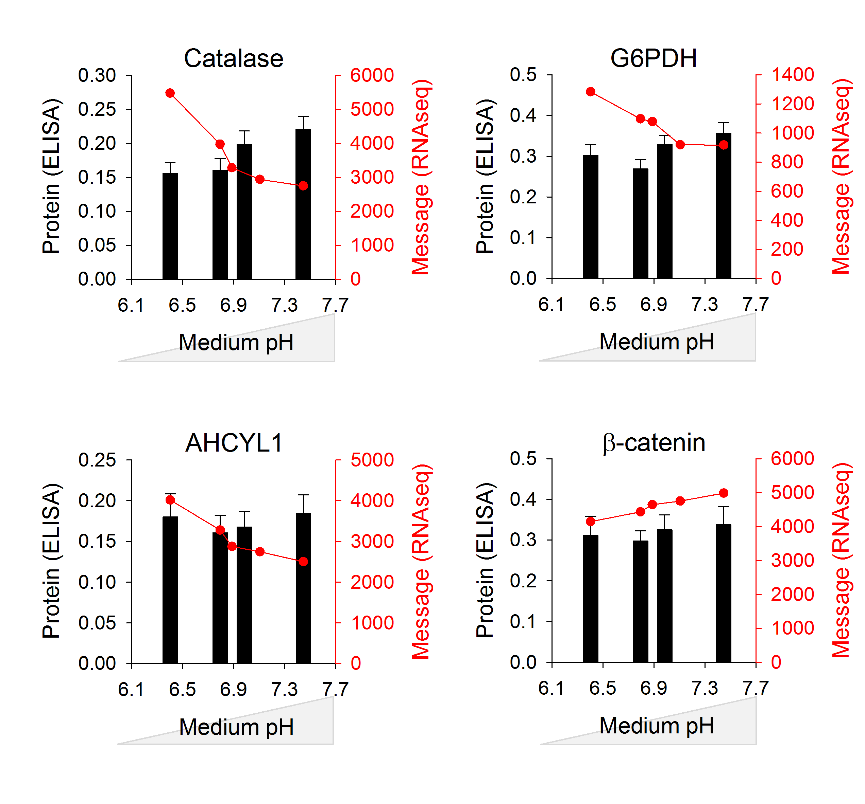
Figure S4: Testing for pH sensitivity of AHCYL1, G6PDH, β-catenin and catalase immuno reactivity.** NRVMs were incubated for 48 hours in medium at pH_e_ between 6.40 and 7.44. ELISA was performed using RIPA total lysates. Graphs show 490nm absorbance values, and they are plotted with results from RNAseq analysis. These four pH-sensitive DEGs were not confirmed at protein level.


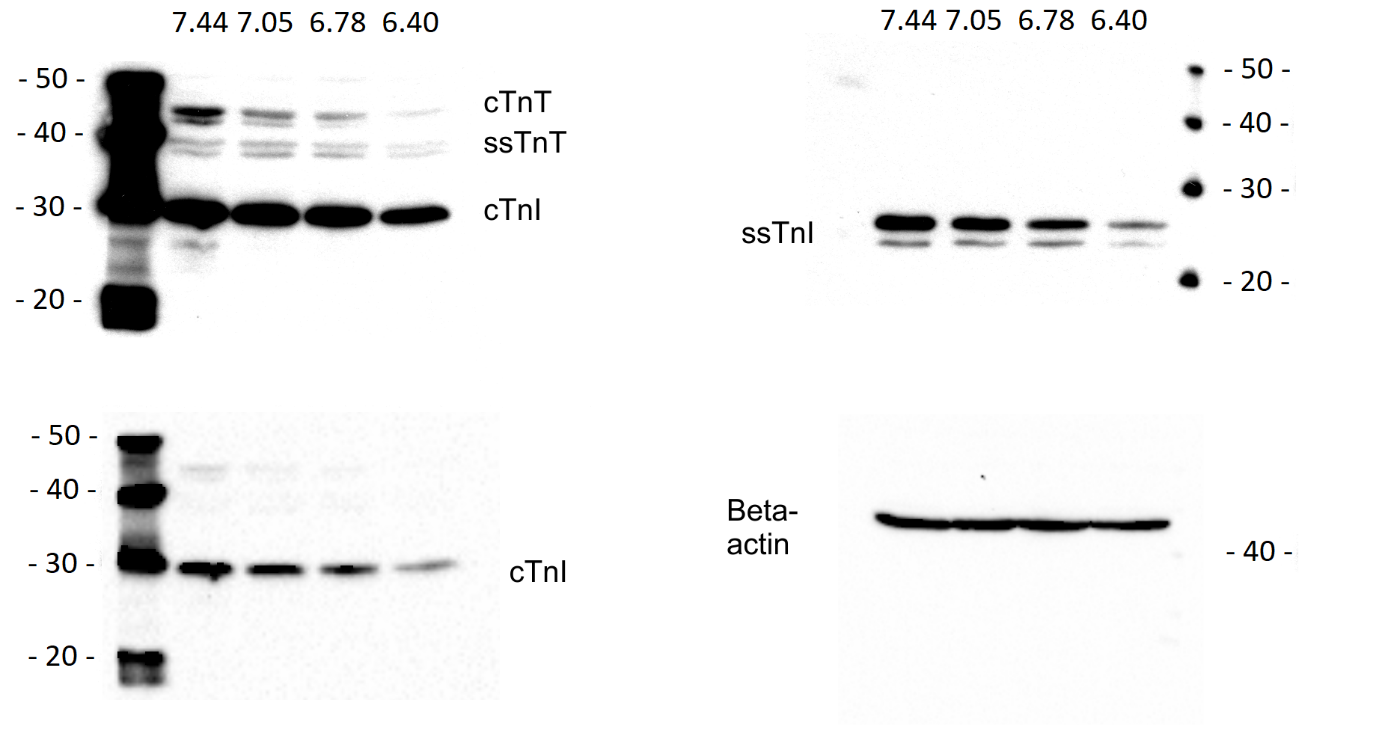


**Figure S5: Uncropped blots for Fig 4A.** The membrane, shown on the left, used to visualize cTnI was also used to probe for cTnT and ssTnT.


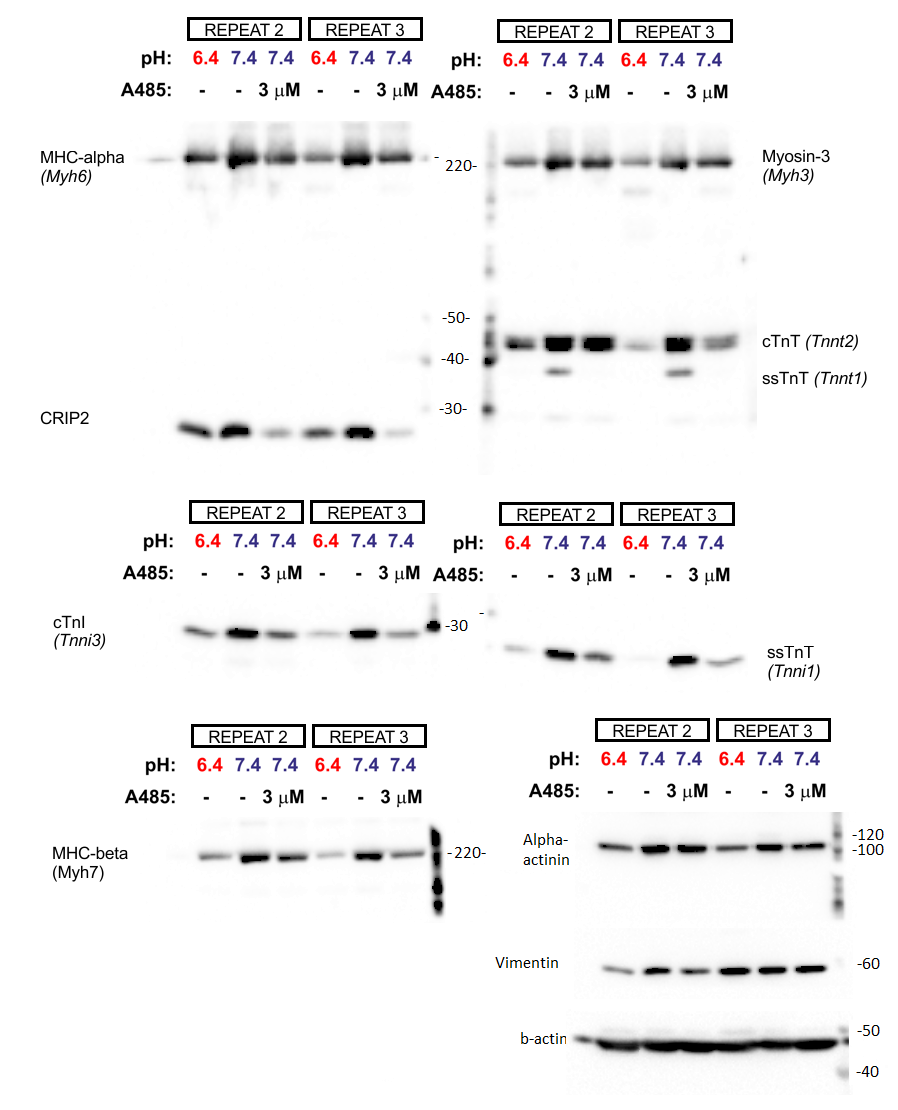


**Figure S6:** Additional repeats of blots for the effect of low pH and p300 inhibition on contractile elements. Repeat 1 is presented in Figure 5. β-actin was re-developed on the membrane that had been used to develop MHC-beta and cTnI. For further information about loading the samples onto gel/membrane see supplementary methods section.

**A**
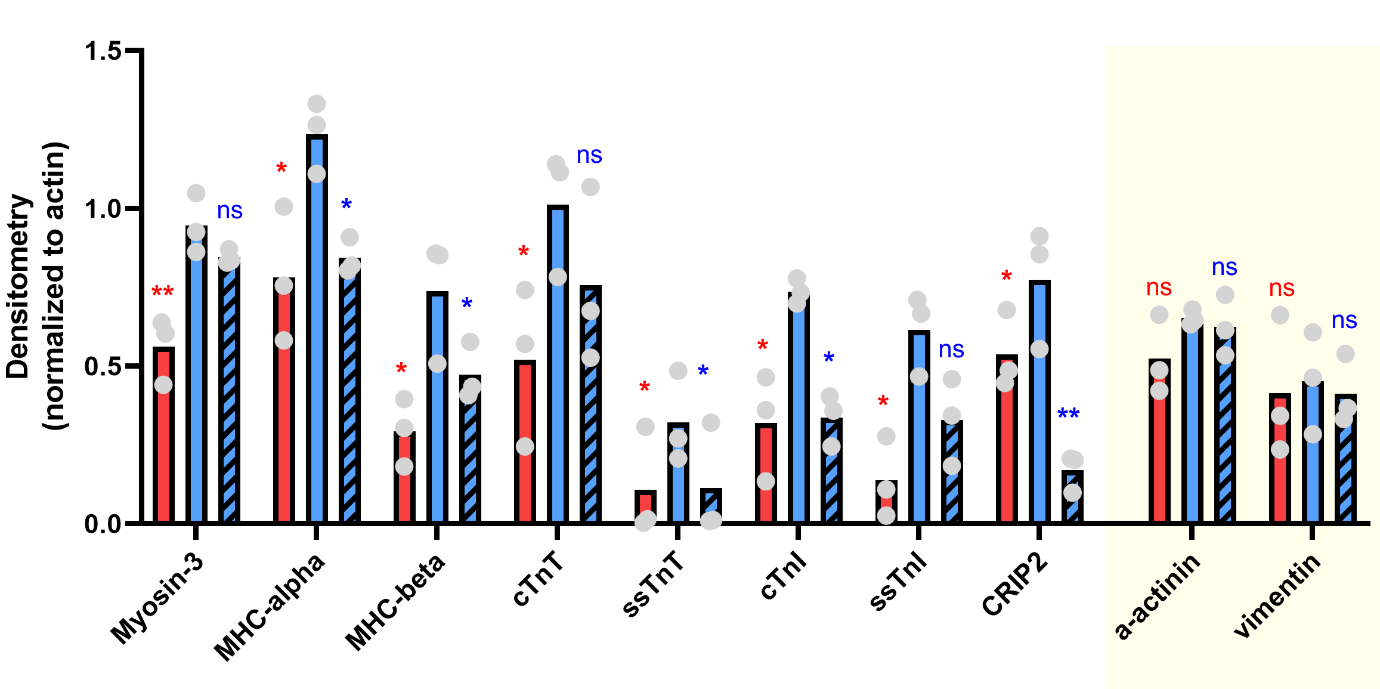


**B**
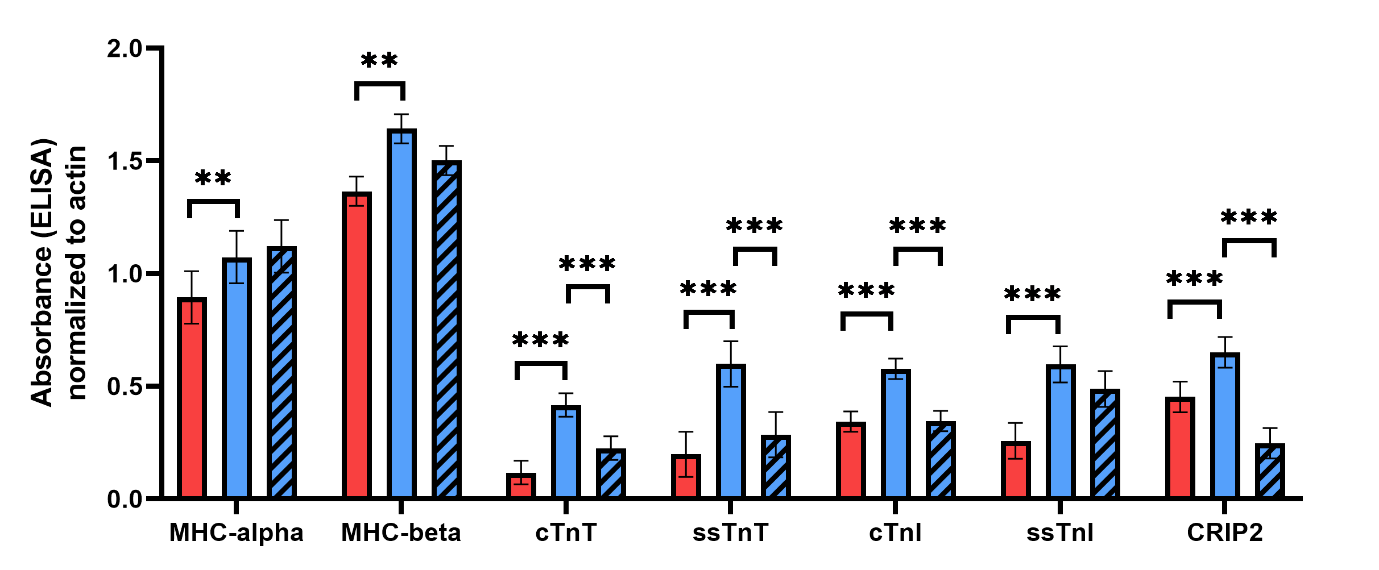


**Figure S7: Densitometric quantification of western blot data.** (A) Densitometry normalized to actin for a range of proteins shown in Fig 5 and S3 (total of 3 independent repeats): incubation at pH 6.4 (red), 7.4 (blue), in the presence (hatched bars) or absence of A458 for 48 h. *P<0.05, **P<0.01. Red asterisks: significant difference at pH 6.4 v 7.4; Blue asterisks: significance effect of A485 (at pH 7.4). Actinin and vimentin are markers of cardiomyocytes and fibroblasts. (B) ELISA measurements of the levels of MHC-alpha, MHC-beta, cTnT, ssTnT, cTnI, ssTnI, CRIP2 protein. The RIPA lysates used for these experiments were from the same batch as the lysates used for immunoblotting (Figure S6). Absorbance was normalized to β-actin signal. Three biological repeats, each with 2-3 technical repeats. Statistical analysis by hierarchical, repeated measures one-way ANOVA.


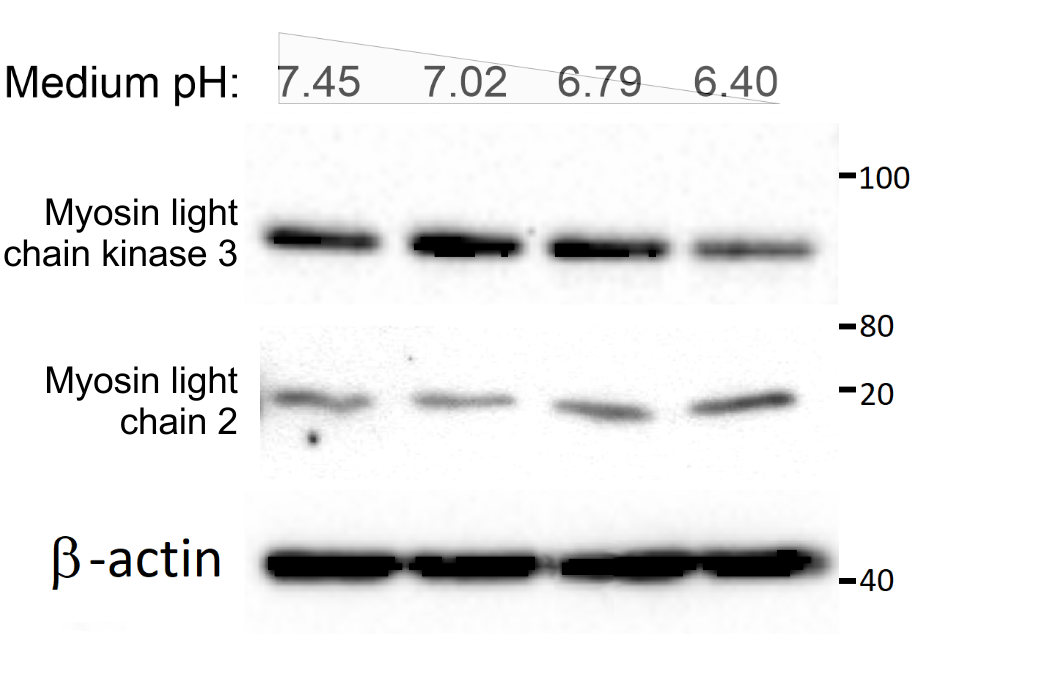


**Figure S8: Testing for pH sensitivity of myosin components.** Western blot analysis of myosin light chain kinase 3 and myosin light chain 2 (*Myl2*), components of the contractile apparatus. Unlike troponin isoforms, the expression of these proteins was determined to be pH insensitive. This indicates that the effect of pH is specifically targeting troponin subunits.

**
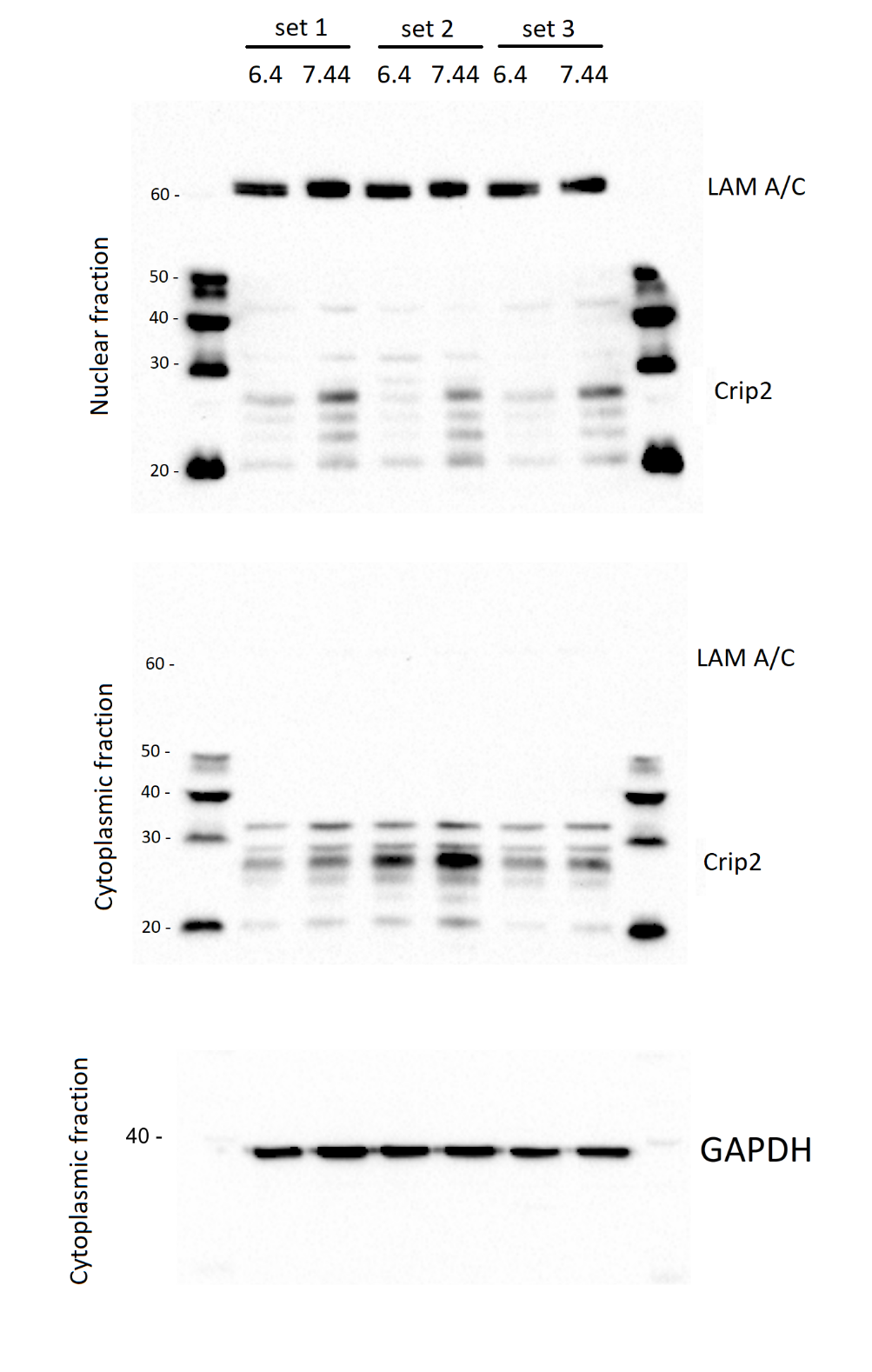
**

**Figure S9: Uncropped blots for Fig 4D.** GAPDH was re-developed on the stripped membrane that had been used probe for cytoplasmic CRIP2.

**
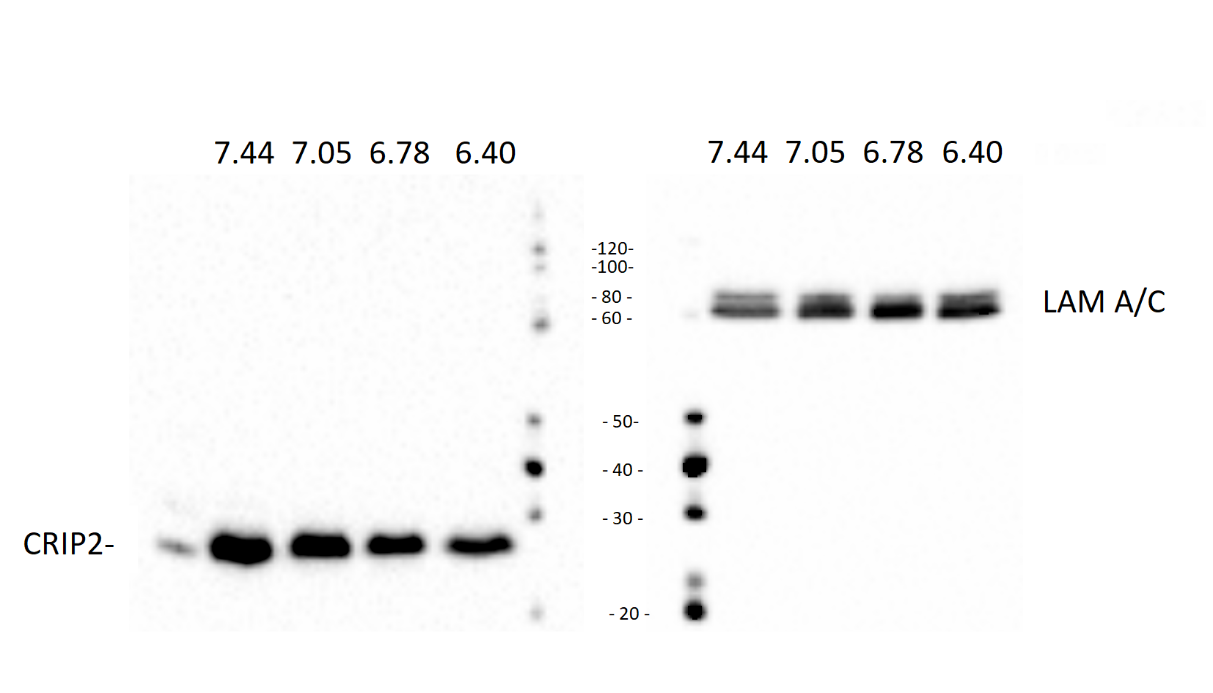
**

**Figure S10: Uncropped blots for Fig 4E.**

**
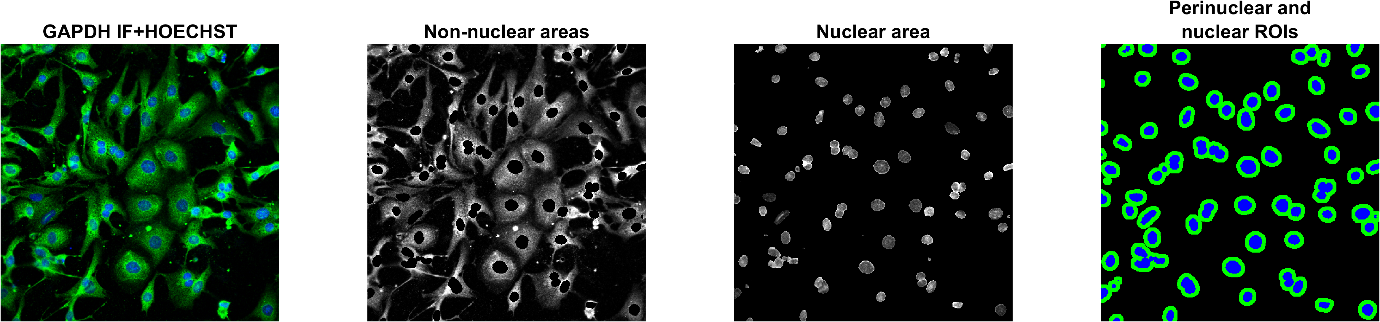
**

**
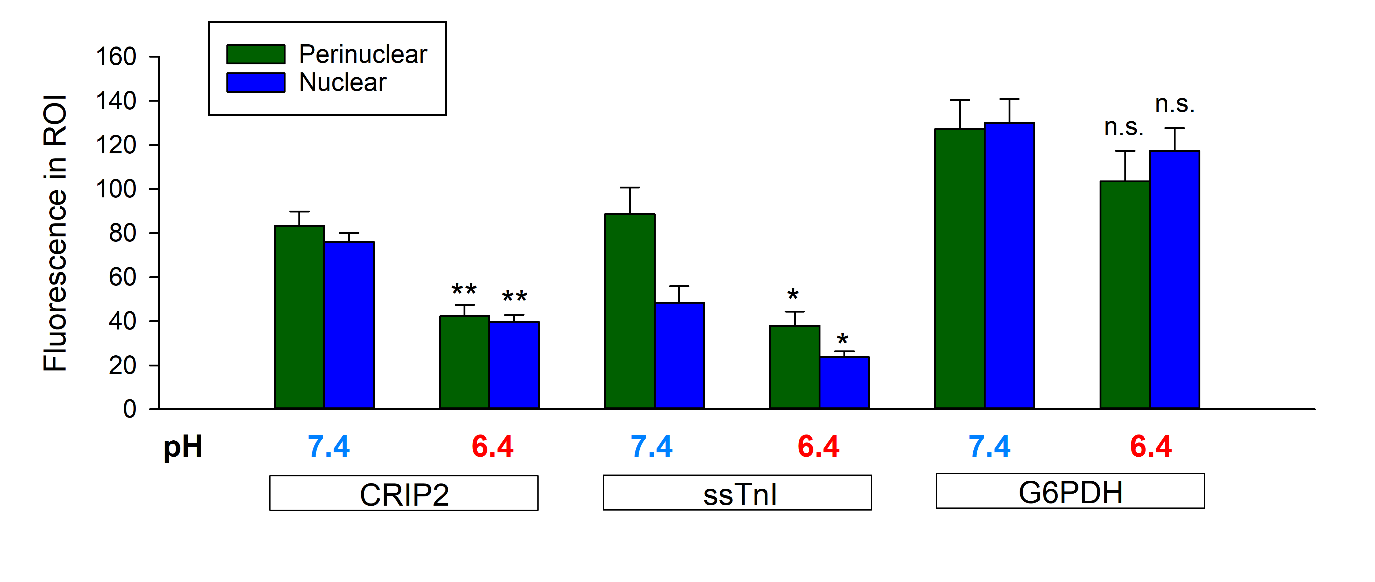
**

**Figure S11: Quantification of immunofluorescence images.** Top panel: imaging method. IF maps were separated into nuclear and non-nuclear regions on the basis of a HOECHST mask. Fluorescence in the nuclear areas and a perinuclear area were calculated, and shown in the bar graphs for three different antibodies (bottom panel). The perinuclear width was set to equal the mean radius of the nucleus. Asterisks: *P<0.05, **P<0.01; comparison between pH 7.4 and 6.4 for each condition.


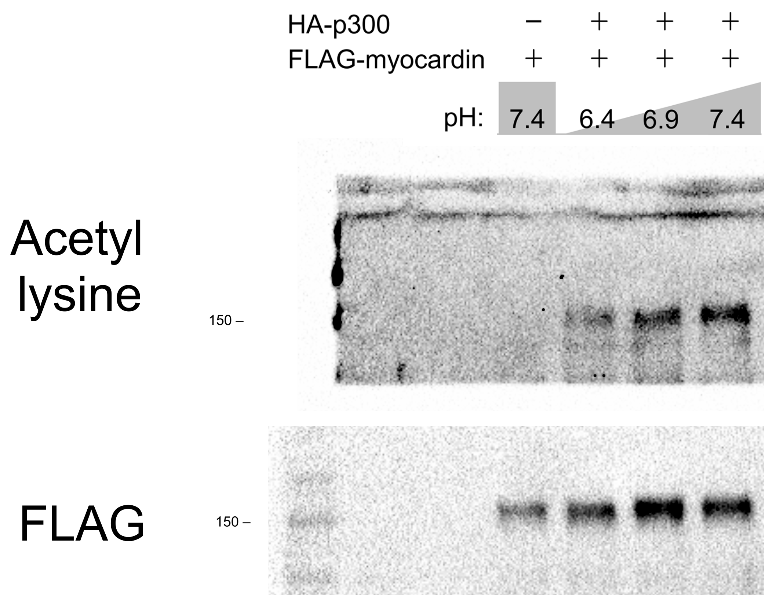


**Figure S12:** Uncropped blots for Fig 5.

**
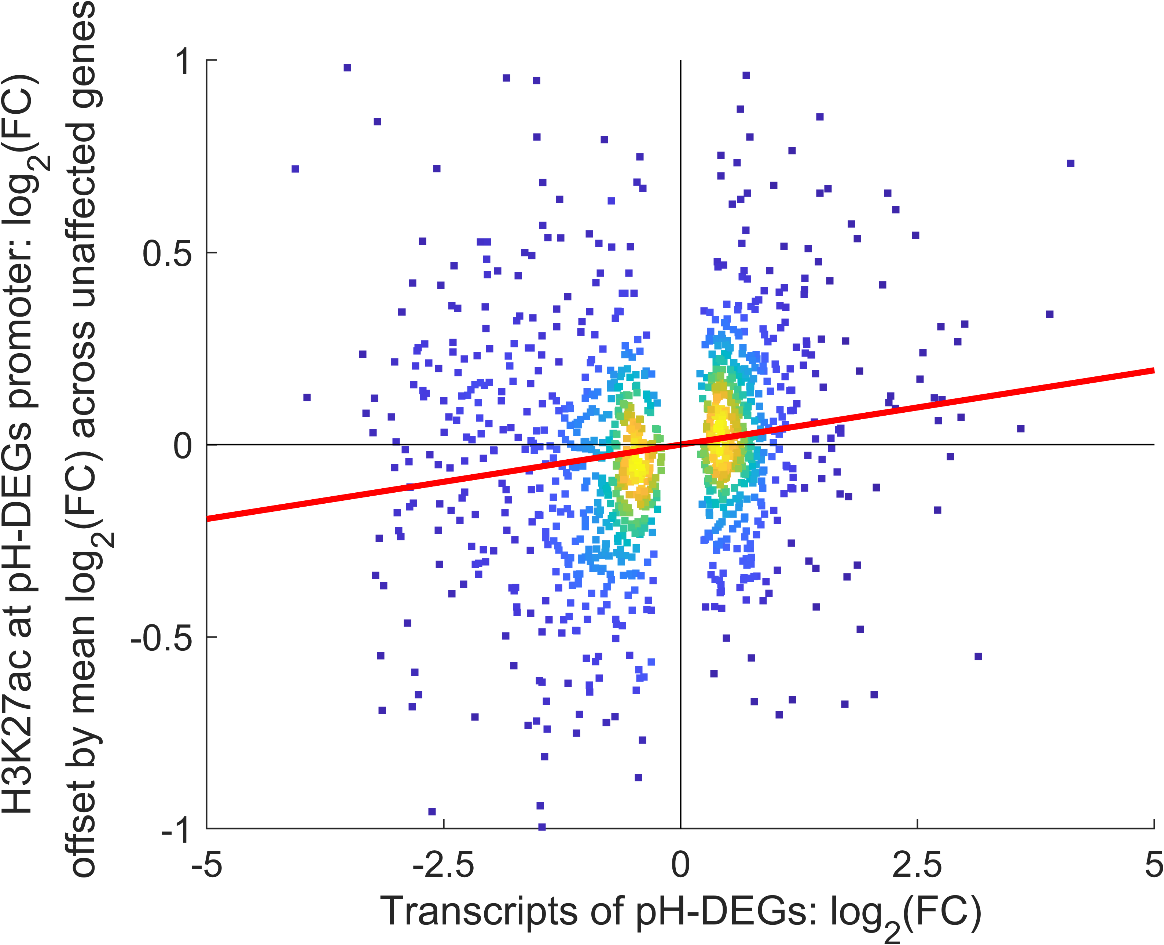
**

**Figure S13: Relationship between transcriptional response of DEGs to pH (x-axis) and change in H3K27ac levels at their promoter, offset to the mean response across all unaffected genes.** Red line shows best fit. Fisher’s exact test: odds-ratio 0.512, P=5×10^-9^.


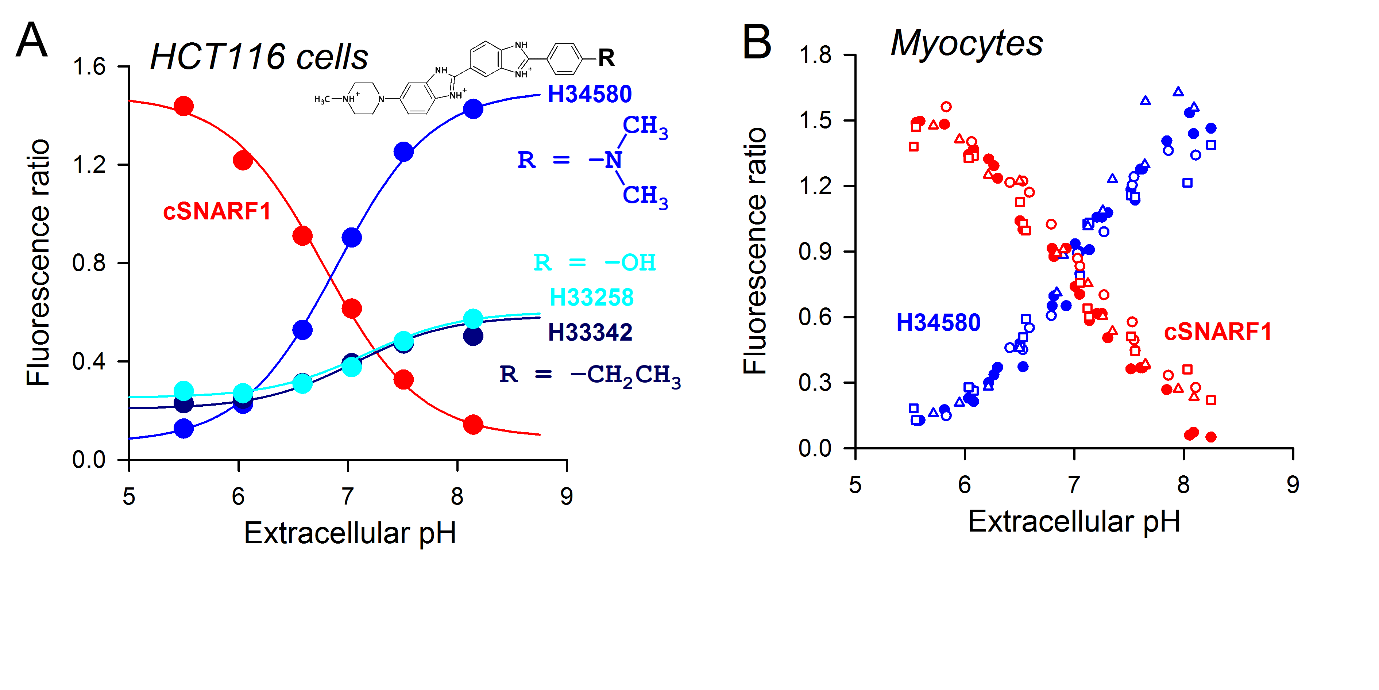


**Figure S14: Identifying pH-sensitive DNA-binding dyes of the Hoechst-family.** (A) Screening various members of the Hoechst family DNA-binding dyes for pH-sensitivity in HCT116 cells superfused with high-K^+^ buffer containing 10 µM nigericin (K^+^/nig). Mean of >600 cells/datapoint. (B) Concurrent calibration of H34580 and cSNARF1 in neonatal ventricular myocytes (NRVMs; filled circles; 5 isolations), adult rat myocytes (empty circles; 4 isolations), adult mouse myocytes (squares; 3 isolations), and adult sheep myocytes (triangles; 2 isolations).


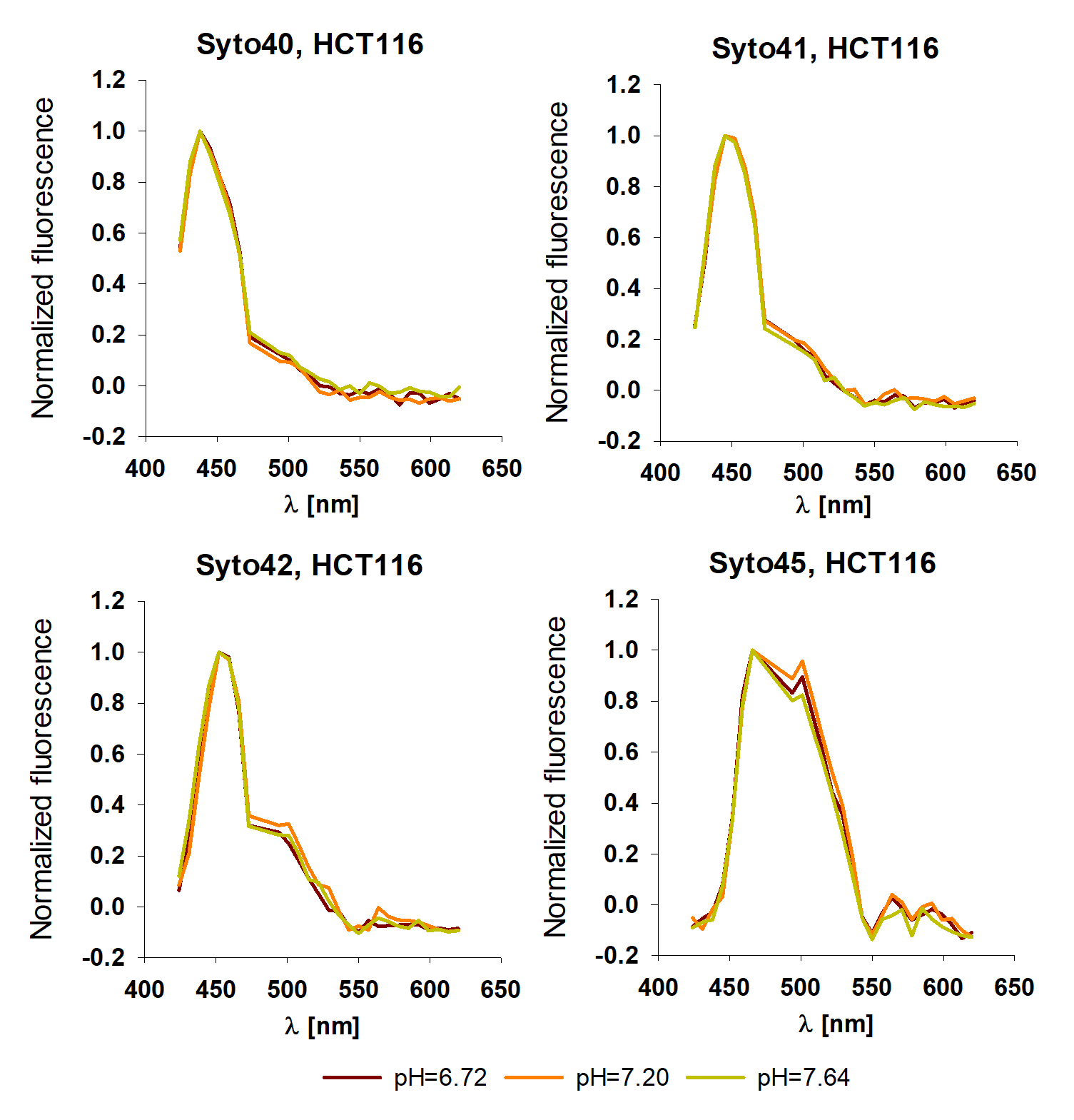


**Figure S15: pH sensitivity of nuclear SYTO dyes**. HCT116 cells were loaded with cell permeable Syto-40, 41, 42 or 45 dyes (Thermo Fisher Scientific, Invitrogen) according to manufacturer recommendations. Cells were superfused at 37°C with high potassium solutions with 10 µM nigericin (140mM KCl, 1MgCl_2_, 0.5mM EGTA, 10mM HEPES or MES, pH set to 6.72, 7.20 or 7.64 at 37°C). Fluorescence spectra of SYTO dyes at three different pH values were obtained at 405 nm excitation and normalized to the maximal value. None of the tested SYTO dyes had meaningful pH sensitivity.


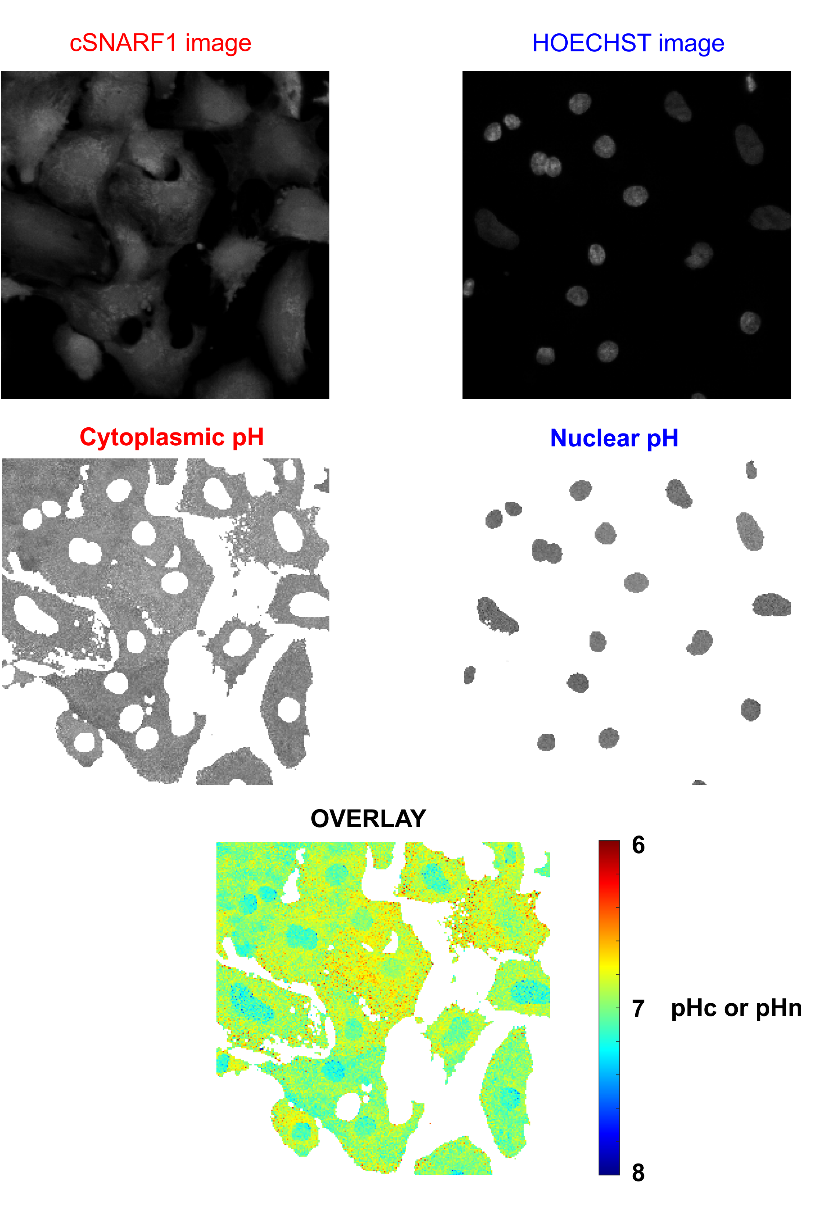


**Figure S16: Example of image processing to obtain cytoplasmic and matching nuclear pH.** NRVM monolayer was loaded with cSNARF1 and HOECHST 34580 and imaged sequentially. The HOECHST image was used to determine a mask defining nuclear regions. This mask then produced an image of cytoplasmic regions and nuclear regions, which can be converted to a ratio, related to pH. The overlay of the nuclear and cytoplasmic pH maps is thus generated.


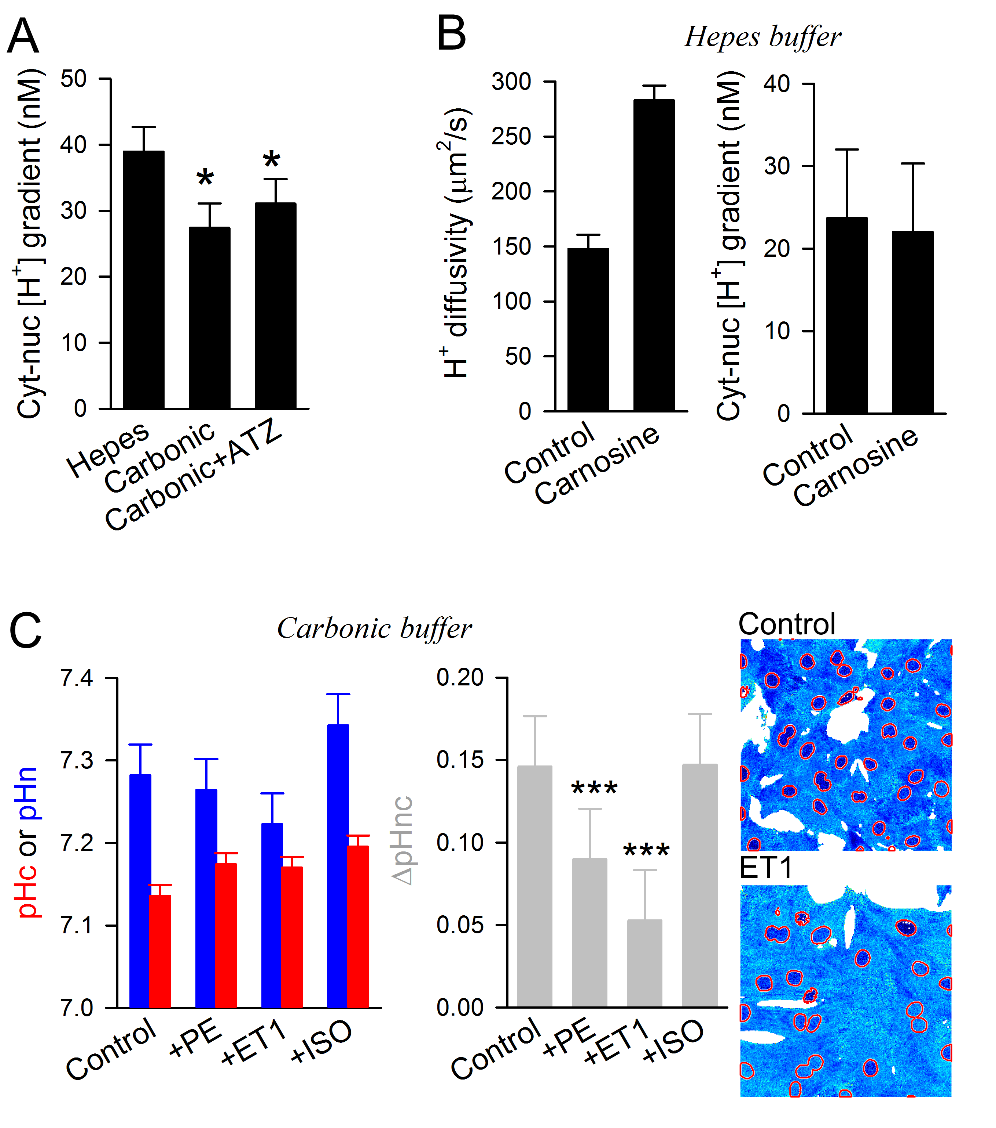


**Figure S17: Characterising the pHn-pHc gradient in NRVMs.** (A) Replacing Hepes with carbonic buffer (5% CO_2_/22mM HCO_3_^-^) in superfusate only modestly decreased cytoplasm-nucleus [H^+^] gradient, which remained alkaline on the nuclear side. Inhibiting carbonic anhydrase with 100 µM acetazolamide (ATZ) had only a modest effect. Mean±SEM from 739-821 cells/3 isolations. (B) Incubation with 30 mM carnosine for 48 hrs increased H^+^ diffusivity measured from the dissipative spread of H^+^ ions uncaged locally by UV light from the donor 6-nitroveratraldehyde. Augmented diffusivity did not dissipate the cytoplasm-to-nucleus [H^+^] gradient, measured in Hepes-buffered conditions. Mean±SEM from 391-476 cells/3 isolations. (C) NRVMs treated with 10 µM phenylephrine (PE), 100 nM endothelin-1 (ET1), or 1 μM isoprenaline (ISO) for 48 hrs. pHn and pHc were measured under superfusion in agonist-free conditions. Mean±SEM from 644-866 cells/4 isolations. Inset shows exemplar images, highlighting nuclei with red boundary.

NHE1 LAMIN A/C HOECHST-33342


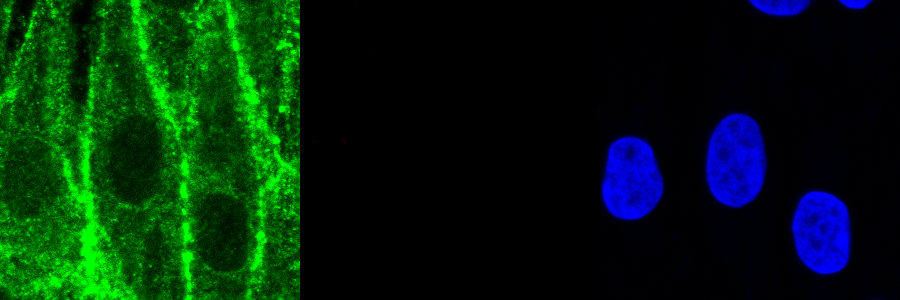


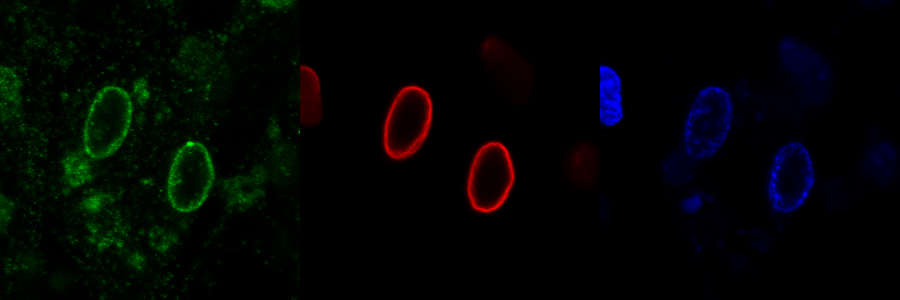


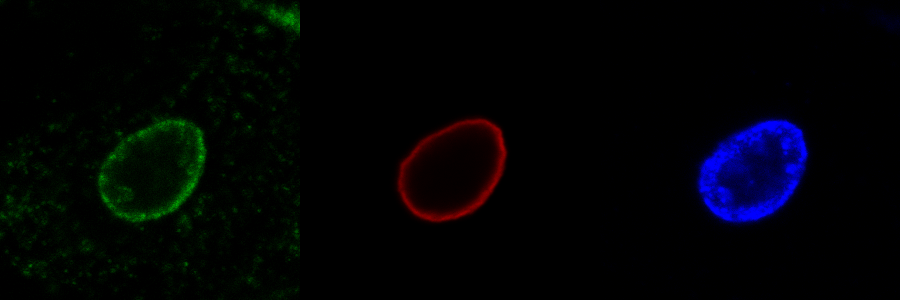


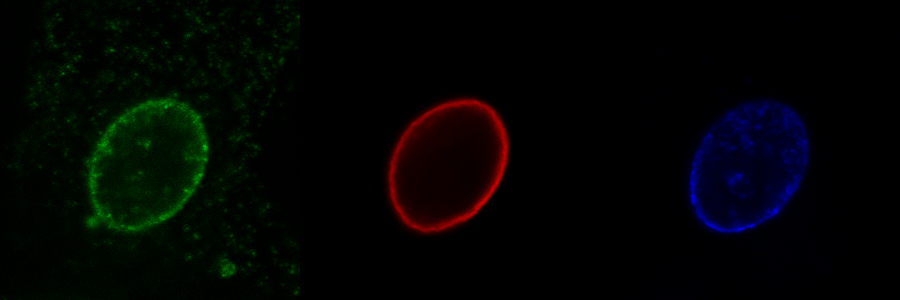


**Figure S18: Immunofluorescence images reproduced from Figure 4I.** Fields of view are 31 µm by 31 µm, 31 µm by 31 µm, 46 µm by 46 µm, 46 µm by 46 µm.
